# Designing Light‐Sensitive Organic Semiconductors with Azobenzenes for Photoelectrochemical Transistors as Neuromorphic Platforms

**DOI:** 10.1002/advs.202509125

**Published:** 2025-07-29

**Authors:** Isabela Berndt Paro, Martina Gini, Francesca D’ Elia, Arianna Massaro, Federica Corrado, Daniela Rana, Ana Varela, Giulia Elli, Matthias Baumann, GiovanniMaria Piccini, Luisa Petti, Daniele Leonori, Ana Belen Muñoz‐García, Michele Pavone, Andreas Offenhäusser, Valeria Criscuolo, Francesca Santoro

**Affiliations:** ^1^ Institute of Biological Information Processing IBI‐3 Forschungszentrum Jülich 52428 Jülich Germany; ^2^ Faculty of Electrical Engineering and IT Neuroelectronic Interfaces RWTH 52074 Aachen Germany; ^3^ Department of Chemical Science University of Naples Federico II, Compl. Univ. Monte Sant'Angelo Via Cintia 21 Naples 80126 Italy; ^4^ Department of Physics “E. Pancini” University of Naples Federico II, Compl. Univ. Monte Sant'Angelo Via Cintia 21 Naples 80126 Italy; ^5^ Institute of Organic Chemistry RWTH Aachen University 52056 Aachen Germany; ^6^ Department of Chemical and Geological Sciences University of Modena and Reggio Emilia Via G. Campi 103 Modena 41125 Italy; ^7^ Institute of Technical and Macromolecular Chemistry RWTH Aachen University 52056 Aachen Germany; ^8^ Sensing Technologies Lab Faculty of Engineering Free University of Bozen‐Bolzano via Bruno Buozzi 1 Bozen‐Bolzano 39100 Italy; ^9^ Present address: Department of Industrial Engineering Università degli Studi di Salerno Fisciano Salerno 84084 Italy

**Keywords:** azobenzenes, optoelectronics, organic bioelectronics, organic neuromorphics

## Abstract

Organic neuromorphic electronics aim to emulate the adaptive behavior of biological synapses using soft, biocompatible materials capable of analog and stimulus‐responsive modulation. While azobenzene‐based semiconductors provide reversible light‐induced switching, their application in mixed ionic‐electronic conductors for neuromorphic systems remains largely unexplored. In this study, photoresponsive organic photoelectrochemical transistors (OPECTs) are engineered by functionalizing PEDOT:PSS with azobenzene derivatives bearing nitro or fluorine substituents. These modifications alter the electronic structure and surface properties of the gate, enabling systematic tuning of interfacial capacitance, a critical parameter governing photogating and neuromorphic response. Optical and electrochemical measurements, supported by DFT calculations reveal that substituent‐dependent modulation of bulk and interfacial capacitance directly impacts gating efficiency. Devices exhibit reversible, analog conductance changes under optical and electrical co‐stimulation, emulating both short‐ and long‐term synaptic plasticity. These results establish a structure–capacitance–function relationship and provide a chemically tunable platform for the development of light‐responsive neuromorphic interfaces in adaptive bioelectronics.

## Graphical abstract

This study focuses on the functionalization of PEDOT:PSS with substituted azobenzenes as gate electrodes and the investigation of the relationship between molecular structure and optoelectronic performance in organic photoelectrochemical transistors (OPECTs). Distinct gate polarization mechanisms during steady‐state and transient operation are demonstrated through gate biasing and light illumination. These findings pave the way for advanced opto‐neuromorphic computing.

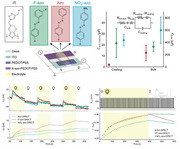

## 1 Introduction

Light as a means of electrical modulation has stimulated increasing interest over the past decades, promoting the development of optoelectronic devices that exhibit low power consumption, high bandwidth, reduced crosstalk, wireless operation, and fast processing and memory functions.^[^ ^1^ ^]^ For instance, recent advances in organic neuromorphic engineering and computing have highlighted the key role of light in the development of artificial neurons and biohybrid synapses.^[^ ^2^, ^3^ ^]^ Beyond computation efficiency, light‐based inputs might be exploited for the emulation of visual perception,^[^ ^4^, ^5^, ^6^ ^]^ integrating information processing and memory functions.^[^ ^2^ ^]^ As a result, optoelectronic synaptic devices have been developed to mimic biological synaptic behaviors, including long‐term plasticity (LTP), which involves persistent changes in the synaptic strength induced by prolonged inputs, and short‐term plasticity (STP), such as paired‐pulse facilitation and depression (PPF and PPD), which modulate synaptic strength based on spiking timing.^[^ ^2^, ^6^ ^]^

In this context, optoelectronic transistors based on organic semiconductors have broadened devices´ functionalities through tailored chemical modifications of photoactive moieties, and a variety of fabrication strategies including blends,^[^ ^7^ ^]^ covalent bonding,^[^ ^8^ ^]^ and surface functionalization.^[^ ^9^ ^]^

In this scenario, exploiting both optical and electrical signals opens opportunities for interfacing electronic devices with biological systems.^[^ ^10^ ^]^ Indeed, optobioelectronics can enable the monitoring, manipulation, and modulation of biological processes ^[^ ^11^, ^12^, ^13^ ^]^ by engineering biocompatible interfaces capable of transducing signals between biological and optoelectronic systems.^[^ ^14^, ^15^ ^]^ Interestingly, optobioelectronic devices can allow for wireless sensing and stimulation with high spatial resolution.^[^ ^16^, ^17^ ^]^ Therefore, to achieve reliable biotic–abiotic integration, operation in aqueous environments is essential.^[^ ^4^ ^]^

A prominent device within this field is the organic photoelectrochemical transistor (OPECT),^[^ ^18^, ^19^ ^]^ which benefits from the properties of organic mixed ionic‐electronic conductors (OMIECs)^[^ ^20^ ^]^ that enable light‐sensitive and electrochemical properties for dynamic interactions with biological systems for biosensing.^[^ ^21^ ^]^ OMIECs are commonly used in OPECTs for their biocompatibility, mechanical flexibility, ease of processing, and ion sensitivity, making them particularly suitable for biological integration as well as neuro‐inspired electronics.^[^ ^22^, ^23^ ^]^

Here, the material selection for the light‐sensitive gate electrode is essential for the rational design of OPECTs, to influence the device´s performance.^[^ ^24^ ^]^ For instance, various photoelectric materials have been employed, such as quantum dots (QDs),^[^ ^19^ ^]^ metal‐organic frameworks (MOFs),^[^ ^25^ ^]^ polyoxometalates (POMs),^[^ ^26^ ^]^ and bioinspired materials.^[^ ^27^, ^28^ ^]^ Light stimulation at the gate might modulate the potential at the electrode/electrolyte interface, resulting in enhanced channel current variation.^[^ ^19^ ^]^ In this context, OMIEC‐based polarizable gate electrodes, which operate in a capacitive (non‐Faradaic) modality and contribute to building up an electrochemical double layer (EDL) capacitance, represent a possible choice for OPECTs thanks to their chemical modification and the exploitation of their volumetric capacitance.^[^ ^24^ ^]^

Within this framework, light‐sensitive molecules such as azobenzenes, known for their light‐responsive *trans‐cis* isomerization,^[^ ^29^ ^]^ can also be exploited due to their tunable chemical, mechanical, and optical properties.^[^ ^30^ ^]^ These molecular switchers are already widely utilized in fields such as photopharmacology ^[^ ^31^ ^]^ drug delivery,^[^ ^32^, ^33^ ^]^ photonics,^[^ ^34^ ^]^ and vision restoration,^[^ ^35^ ^]^ and now show promise for neuromorphic systems.^[^ ^36^, ^37^ ^]^

A significant recent advancement in gate electrode engineering involves the functionalization of poly(3,4‐ethylenedioxythiophene) polystyrene sulfonate (PEDOT:PSS) through click chemistry, incorporating azobenzene derivatives.^[^ ^38^ ^]^ This approach enables the precise coupling of various functional compounds onto a methylene azide PEDOT:PSS derivative (N_3_‐PEDOT:PSS), imparting diverse properties to PEDOT:PSS‐based electrodes in bioelectronics.^[^ ^39^, ^40^ ^]^

Furthermore, azobenzene‐based OPECTs have demonstrated synaptic plasticity, memory/erasing neuromorphic functions, and the ability to mimic neural processes, particularly when N_3_‐PEDOT:PSS is coupled with alkyne‐substituted azobenzenes. This yields photo‐responsive conductive polymers with tailored properties.^[^ ^41^ ^]^

Despite these advancements, a key challenge remains: the relationship between the structure and composition of functional molecules and their influence on the gating effect in OPECTs is still not completely understood. Gaining deeper insight into this relationship is critical for optimizing device performance and expanding its potential applications in areas such as biosensing, neuromorphic computing, and related fields.

In this study, we explore how substitutions on azobenzenes influence the optoelectronic response of X‐azo‐tz‐PEDOT:PSS films when employed as gate materials. We selected functional groups that either tune the optical properties, such as the nitro group (─NO_2_),^[^ ^29^ ^]^ or modify the electrode's surface polarity by introducing an electronegative atom, such as fluorine.^[^ ^42^, ^43^ ^]^ Comprehensive characterization of the optical and electrochemical properties of the various gate materials revealed distinct optoelectronic behaviors, depending on the nature of the light‐sensitive moiety. UV–vis spectroscopy, cyclic voltammetry (CV), and electrochemical impedance spectroscopy (EIS) were used to assess how functionalization affects light absorption and capacitance, both of which strongly influence gating efficiency in planar OECT configurations. By integrating these materials into OPECTs, we examined how the steady‐state responses under dark and illuminated conditions correlate with optical and electrochemical properties, particularly in terms of gate polarization and gating efficiency. Additionally, density functional theory (DFT) calculations provided insight into the electronic states governing these behaviors. Finally, we evaluated the potential of these materials for memory and neuromorphic computing applications using transient optical and electrical pulsed stimulation, evaluating synaptic conditioning and extinction.

## 2 Results and Discussion

**Figure 1:**
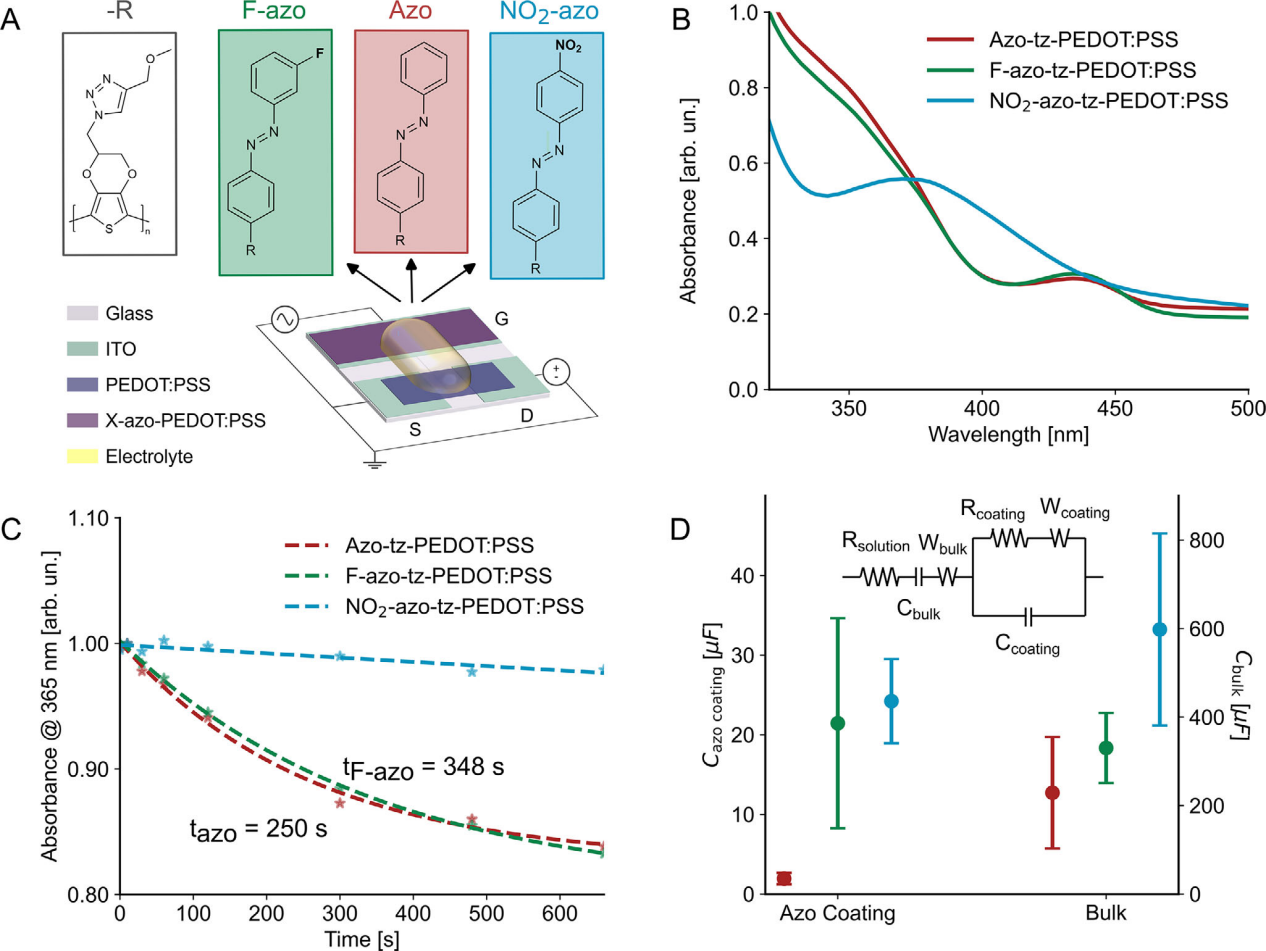
Schematics and properties of the proposed OPECT. A) Schematic representation of the OPECT architecture comprising a photo‐responsive gate functionalized with azobenzene‐based molecules and a spin‐coated PEDOT:PSS channel. B) UV–vis absorbance spectra of the gate electrodes functionalized with three different azobenzene derivatives. C) Time‐dependent evolution of the absorbance spectra upon UV illumination (λ = 365 nm, intensity = 0.31 mW cm^−2^), showing the photoisomerization behavior of the azobenzene‐functionalized gates. D) Surface and bulk capacitance of the functionalized films, extracted by fitting impedance spectroscopy data acquired in dark using the equivalent circuit shown in the inset.

The structure of the proposed OPECTs is illustrated in **Figure** **1**A. Here, the source and drain electrodes are connected by a PEDOT:PSS channel, as described in the Experimental Section.^[^ ^41^ ^]^ The gate electrodes consist of electrodeposited N_3_‐PEDOT:PSS films, from an aqueous solution of EDOT‐N_3_ (Figure S1A, Supporting Information) and PSSNa, functionalized via click chemistry with azobenzene derivatives bearing alkyne side groups. Specifically, three azobenzene derivatives were selected: an unsubstituted azobenzene (azoalkyne, Figure S1B, Supporting Information), and two azobenzenes substituted with electronegative and electron‐withdrawing (EWD) groups. One derivative included a nitro group (NO_2_‐azoalkyne, Figure S1C, Supporting Information) at the para position, and another with a fluorine atom (F‐azoalkyne, Figure S1D, Supporting Information) at the meta position relative to the diazene group (─N═N─). EWD groups like ─NO_2_, when positioned para to the diazene group, extend π‐conjugation through mesomeric effects, resulting in a bathochromic shift of the π–π* absorption band, which overlaps with the n–π* transition. In contrast, meta‐positioned substituents, such as fluorine, do not contribute to delocalization across the entire azobenzene π system and therefore do not significantly shift the absorption band. However, fluorine's strong electronegativity may increase the polarity of the azobenzene structure, potentially influencing the gate/electrolyte interface.

Covalent linking the photo‐responsive molecules to the gate surface led to the formation of the corresponding polymers: azo‐tz‐PEDOT:PSS, F‐azo‐tz‐PEDOT:PSS, and NO_2_‐azo‐tz‐PEDOT:PSS, hereafter referred to as azo, F‐azo, and NO_2_‐azo, respectively. Fourier Transform Infrared (FT‐IR) spectroscopy confirmed successful functionalization of N_3_‐PEDOT:PSS as evidenced by a reduction in the ─N_3_ stretching band at 2097 cm^−1^ (Figure S2, Supporting Information). The optical properties of the functionalized gate electrodes were examined using UV–vis spectroscopy (Figure 1B). The absorbance spectra for azo and F‐azo films exhibited similar profiles, with primary peaks at 338 and 344 nm, respectively, and secondary peaks at 436 nm. In contrast, the NO_2_‐azo film exhibited a single dominant peak at 372 nm. These results are consistent with the spectra of the corresponding azo‐alkynes in THF solution (Figure S3, Supporting Information), featuring a strong π–π^*^ transition at shorter wavelengths and a weaker n–π^*^ transition at longer wavelengths.^[^ ^30^ ^]^

Upon UV light irradiation, azo and F‐azo films showed reduced absorbance at 365 nm, attributed to *trans‐*to*‐cis* isomerization, whereas the NO_2_‐azo films showed minimal change over time (Figure 1C; Figure S4, Supporting Information), likely due to simultaneous to *trans‐*to*‐cis* and *cis‐*to*‐trans* isomerization under the same wavelength.^[^ ^30^ ^]^ Time‐resolved absorbance spectra further revealed that the azo film reached maximum *trans*‐to‐*cis* conversion within 250s, while the F‐azo film required 348 s to achieve similar saturation (Figure 1C).

To elucidate the effect of the substituents on the gate/electrolyte interface, the surface of the gate electrodes was characterized using atomic force microscopy (AFM) and contact angle measurements. Surface roughness ranged from 16 to 32 nm, with F‐azo films being the smoothest (Figure S5, Supporting Information). Azo films were more hydrophobic compared to NO_2_‐azo and F‐azo films (Table S1, Supporting Information).

Then, being the gating efficiency of optoelectronic transistors correlated to the capacitance of the gate that can be light‐modulated,^[^ ^24^, ^44^ ^]^ the electrochemical properties of the different materials were investigated via electrochemical impedance spectroscopy (EIS) in the dark and under illumination. In dark conditions, azo films exhibited the lowest capacitance, while F‐azo films had the highest (Figure S6, Supporting Information). A similar trend was observed in the impedance amplitudes: azo films had the highest values and F‐azo the lowest (Figure S7, Supporting Information).

Here, the impedance spectra were fitted using the equivalent circuit in Figure 1D, including R_solution_, (resistance of the electrolyte and electrical contacts), C_bulk_ (bulk capacitance of the N_3_‐PEDOT:PSS), and a parallel of R_coating_ and C_coating_, representing charge transfer resistance and interfacial barrier properties, respectively.^[^ ^45^ ^]^ Warburg elements (W_bulk_ and W_coating_) were also included to account for non‐idealities in the conductive polymer films, such as surface roughness and non‐uniform ion diffusion.^[^ ^46^ ^]^ Bulk capacitance ranged from 250 µF (azo and F‐azo) to 600 µF (NO_2_‐azo), while surface (coating) capacitance increased from ≈3 µF (azo) to ≈25 µF (NO_2_‐azo and F‐azo) (Figure 1D). These differences suggest that substituents significantly influence the electrochemical properties, likely due to differences in electronegativity, for instance, where fluorine may induce a strong dipole formation at the electrode surface.^[^ ^47^ ^]^

To further explore these effects, the influence of light illumination on the different materials was investigated by varying both intensity (0.92, 2.96 and 4.73 mW cm^−2^, corresponding to the 20%, 60% and 100% of source power, respectively, Table S2, Supporting Information) and exposure time (2 and 6 min), the latter chosen to discriminate *trans*‐to‐*cis* isomerization from charge transfer effects. Based on the calculated doses (Table S2, Supporting Information), isomerization is expected to reach completion within two minutes for all devices.

The most significant changes occurred in N_3_‐PEDOT:PSS bulk (Figure S8B,D,F, Supporting Information), compared to the surface coatings (Figure S8A,C,E, Supporting Information). Here, in F‐azo and NO_2_‐azo films, the bulk capacitance reached its maximum after 2 min of illumination at 2.96 mW cm^−2^, reaching 300 and 350 µF, respectively. In contrast, the azo film showed a less distinct saturation trend, with a modest increase (2.5 µF after 2 min at 0.92 mW cm^−2^).

On the other hand, surface capacitance was more influenced by exposure time than intensity across all films, increasing by ≈30 µF (azo) and ≈20 µF (F‐azo and NO_2_‐azo) after 6 min at 2.96 mW cm^−2^. Control experiments confirmed that this modulation only occurs with azobenzene functionalization (Figure S8G,H, Supporting Information).

Additionally, cyclic voltammetry (CV) in the dark (Figure S9, Supporting Information) revealed that the azobenzene oxidation peak was unaffected by the ─NO_2_ substitution but absent in F‐azo films (Figure S9B, Supporting Information). The diazene group's reduction peak shifted to higher potentials in F‐azo and lower in NO_2_ ‐azo, the latter due to nitro group reduction.^[^ ^48^ ^]^ Under light illumination (0.92, 2.96, and 4.73 mW cm^−2^) with exposure times of 2 and 6 min, only azo and F‐azo films showed reduced hysteresis, attributed to *cis*‐azobenzene conformation. NO_2_‐azo did not show this, likely due to concurrent isomerization; N_3_‐PEDOT:PSS showed no photochemical response (Figure S9D, Supporting Information).

**Figure 2:**
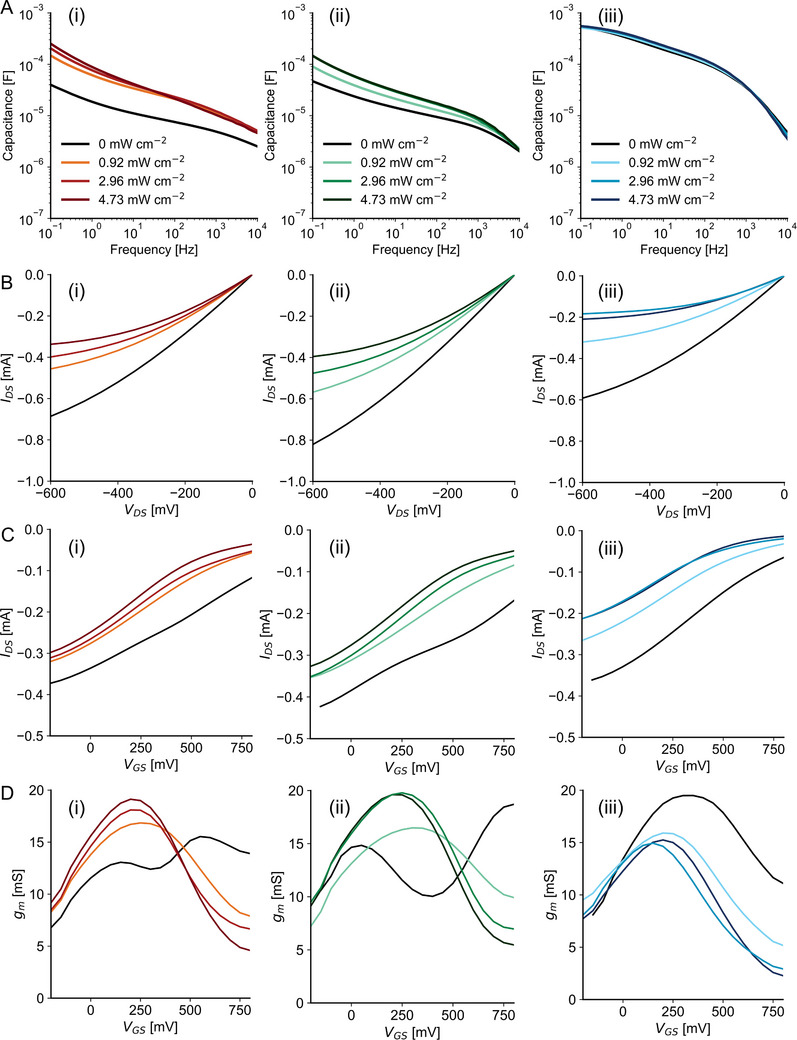
Capacitance and steady‐state characterization of OPECTs. A) Capacitance of azo (i), F‐azo (ii) and NO_2_‐azo (iii) films, calculated from EIS measurements in dark (0 mW cm^−2^) and after 6 min of illumination at different light powers (0.92, 2.96 and 4.73 mW cm^−2^). B) Output (V_GS_ = 300 mV) and C) transfer (V_DS_ = −200 mV) curves of the OPECTs with azo (i), F‐azo (ii) and NO_2_‐azo gates (iii), in dark (black curve) and at different illumination powers (0.92, 2.96 and 4.73 mW cm^−2^). D) Transconductance at V_DS_ = −200 mV, with azo (i), F‐azo (ii), and NO_2_‐azo gates (iii), in dark (black curve) and at different illumination powers (0.92, 2.96, and 4.73 mW cm^−2^). All OPECTs were previously illuminated for 5 min with the corresponding light power.

Subsequently, the performance of the OPECTs was characterized, with a focus on the effect of the substituents on gate polarization.^[^ ^41^ ^]^ Measurements were carried out under steady‐state conditions in the dark and under varying UV‐light exposure (**Figure** **2**; Figures S10 and S11, Supporting Information). To assess the effect of light intensity on device behavior, the samples were pre‐illuminated for 2 or 5 min before measurements.

Output curves recorded at a fixed gate‐source voltage (V_GS_ = 300 mV) (Figure 2B) and transfer curves at a fixed drain‐source voltage (V_DS_ = −200 mV) (Figure 2C) showed that all OPECTs exhibited light‐induced channel current modulation, with current increasing proportionally to light intensity across all gate materials. Transconductance, evaluated at a fixed V_DS_ = −200 mV, is reported in Figure 2D. Under dark conditions, NO_2_‐azo‐OPECTs demonstrated the highest gating efficiency, which progressively decreased with increasing light intensity, even at low light intensities, eventually converging with the values observed for the other devices.

In contrast, azo‐ and F‐azo‐OPECTs displayed a distinct transconductance profile, characterized by a valley centered around a gate bias of 300–400 mV. Unlike the NO_2_‐azo devices, these showed enhanced gate efficiency under illumination. To further investigate the effect of light on gate polarization and efficiency, transconductance values at V_GS_ = 300 mV and V_DS_ = −200 mV, were plotted as a function of applied light intensity (Figure S11A, Supporting Information). After illumination, all devices, regardless of gate composition, exhibited similar transconductance responses across the range of light intensities. The on/off current ratio (Figure S11B, Supporting Information) followed a comparable trend, with all devices reaching similar ratios at 2.96 mW cm^−2^, while NO_2_‐azo‐OPECTs achieved their peak ratio at 0.92 mW cm^−2^. Control experiments using N_3_‐OPECTs (unfunctionalized gate electrode) showed no changes in behavior upon light exposure (Figure S10A, Supporting Information), confirming that the observed effects arise specifically from the azobenzene‐based gate functionalization.

**Figure 3:**
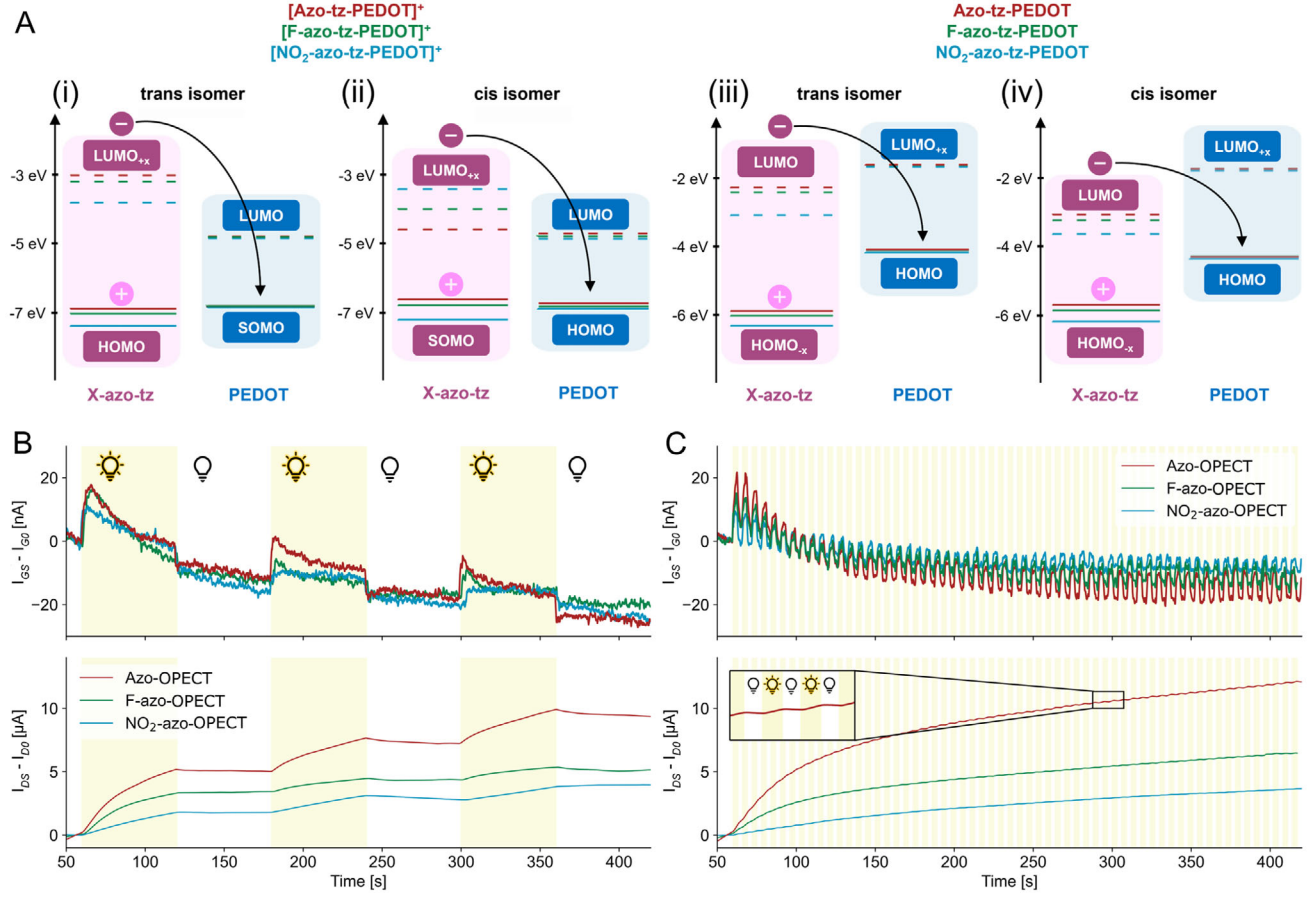
Computational analysis and pulsed light gate bias. A) Energy levels of *trans* (i) and *cis* (ii) of oxidized, biased, [X‐azo‐tz‐PEDOT]^+^ compounds and of *trans* (iii) and *cis* (iv) of the neutral, unbiased, system obtained from DFT‐TDDFT calculations: HOMO/LUMO orbitals from azobenzene and PEDOT moieties are highlighted in pink and blue, respectively. Gate (top) and channel (bottom) currents recorded during B) slow (1 min of ON time) and C) fast (3 s of ON time) light stimulation, with V_GS_ = 0 V and V_DS_ = ‐200 mV. Offset currents were removed to improve the comparison between the device's responses. Yellow background indicates periods with light ON (2.96 mW cm^−2^, 365 nm).

To further comprehend the influence of the substituents on the mechanisms observed under electrical and photo‐induced gating, density functional theory (DFT) and time‐dependent density functional theory (TD‐DFT) calculations were conducted on model systems of the X‐azo‐tz‐PEDOT compounds in both neutral (unbiased) and oxidized (biased) states (Figure 3A; Figure S13, Supporting Information). For all three materials, the energy level alignment was evaluated for both *trans* and *cis* conformers of the azobenzene moieties and the PEDOT backbone.

Under an applied positive bias (300 mV), the system is modeled in its oxidized form, denoted as [X‐azo‐tz‐PEDOT]^+^. In the *trans* configuration, the HOMO levels of azo (−6.95 eV) and F‐azo (−7.08 eV) closely aligned with the PEDOT SOMO (−6.86 and −6.87 eV, respectively), suggesting possible charge trapping. In contrast, NO_2_‐azo exhibits a lower HOMO (−7.40 eV), minimizing overlap with the PEDOT SOMO. This alignment in azo and F‐azo may promote charge trapping, contributing to the reduced gate efficiency observed ≈300 mV in the transconductance curves under dark conditions (Figure 2A‐i‐ii), whereas NO_2_‐azo avoids this trapping, resulting in enhanced gating performance (Figure 2A‐iii).

In the *cis* configuration, energy alignments shift: for azo and F‐azo, the SOMO is localized on the azobenzene moiety (−6.65 and −6.81 eV, respectively), while the HOMO resides on the PEDOT backbone (−6.76 and −6.84 eV). In NO_2_‐azo, the energy levels remain largely unchanged (HOMO: −7.19 eV; SOMO: −6.90 eV; **Figure** **3A‐ii**).

LUMO levels also vary among the materials, with NO_2_‐azo showing the highest (−3.71 eV) and azo the lowest (−4.79 eV). Upon illumination, electrons are excited into the azo LUMOs and can be transferred to the PEDOT HOMO/SOMO levels. This charge transfer is less efficient in NO_2_‐azo due to the larger energy gap, which accounts for its diminished gating efficiency under illumination. In contrast, azo and F‐azo films exhibit energy alignments that mitigate the gating suppression ≈300 mV, explaining their improved performance under light exposure (Figure 2D). In the absence of an applied bias, the neutral X‐azo‐tz‐PEDOT systems exhibit energy level alignments consistent with spectroscopic UV–vis data. The *trans* forms of azo and F‐azo display similar HOMO (−5.88 and −6.01 eV) and LUMO (−2.32 and −2.47 eV) levels, while NO_2_‐azo shows lower values (HOMO: −6.30 eV; LUMO: −3.12 eV) and a reduced band gap (**Figure** **3**A‐iii). The *cis* isomers exhibit narrower HOMO–LUMO band gaps: azo (−5.59, −3.04 eV), F‐azo (−5.75, −3.19 eV), and NO_2_‐azo (−6.06, −3.58 eV). The HOMO (−4.14−4.21 eV) and LUMO (−1.69−1.76 eV) levels of the PEDOT backbone remain largely unchanged across configurations (Figure 3A‐iv).

In both *trans* and *cis* states, light‐induced current arises from electron transfer from the azo‐LUMO to the PEDOT‐HOMO. As previously reported,^[^ ^41^ ^]^ this process can be initiated by both isomers, particularly under conditions of incomplete photoisomerization. Despite the favorable alignment of NO_2_‐azo with the PEDOT‐LUMO, its lower photogating efficiency under illumination may be attributed to the competing *trans*–*cis* and *cis*–*trans* isomerization processes at 365 nm, which could limit net gate charge generation. This effect likely contributes to the reduced channel modulation observed for NO_2_‐azo compared to azo and F‐azo films under illumination.

To verify the neuromorphic capabilities of the X‐azo‐OPECTs, synaptic plasticity (namely time‐dependent modulation of channel conductance) was investigated by applying sequential light stimulation and combined light/electrical stimulation to the gate terminal (pre‐synaptic end) while monitoring the output) channel current (post‐synaptic end).

The photo‐induced gating effect was then investigated across the different gate materials under illumination, without applying an electrical gate bias (V_GS_ = 0 V, V_DS_ = −200 mV). Two light stimulation regimes were tested: a slow‐stimulation mode (120 s period, 50% duty cycle, Figure 3B) and a fast‐stimulation mode (6 s period, 50% duty cycle, Figure 3C), each with a total illumination time of 3 min and a light intensity of 2.96 mW cm^−2^. The OPECTs exhibited a typical gating response observed in light‐responsive transistors: upon illumination, a small gate current in the nanoampere range was generated, resulting in modulation of the channel current into the microampere range, corresponding to a gain of ≈10^3^.^[^ ^28^ ^]^ This behavior was consistent across both stimulation regimes (Figure S12, Supporting Information). In the slow‐stimulation mode, the charge accumulated at the gate electrode was calculated and found to be comparable for all three materials (Figure S12A, Supporting Information), with similar amplification of the channel current, expressed as a percentage increase in conductance (Figure S12B, Supporting Information). This demonstrates that the light‐induced gate polarization can be exploited to achieve synaptic plasticity in X‐azo‐OPECTs. In particular, this can be considered as long‐term potentiation since the effect on the channel conductance persists even after removal of the stimulus. A similar result is obtained in the fast‐stimulation mode, with the NO_2_‐azo‐OPECTs exhibiting lower potentiation (Figure S12B, Supporting Information).

**Figure 4:**
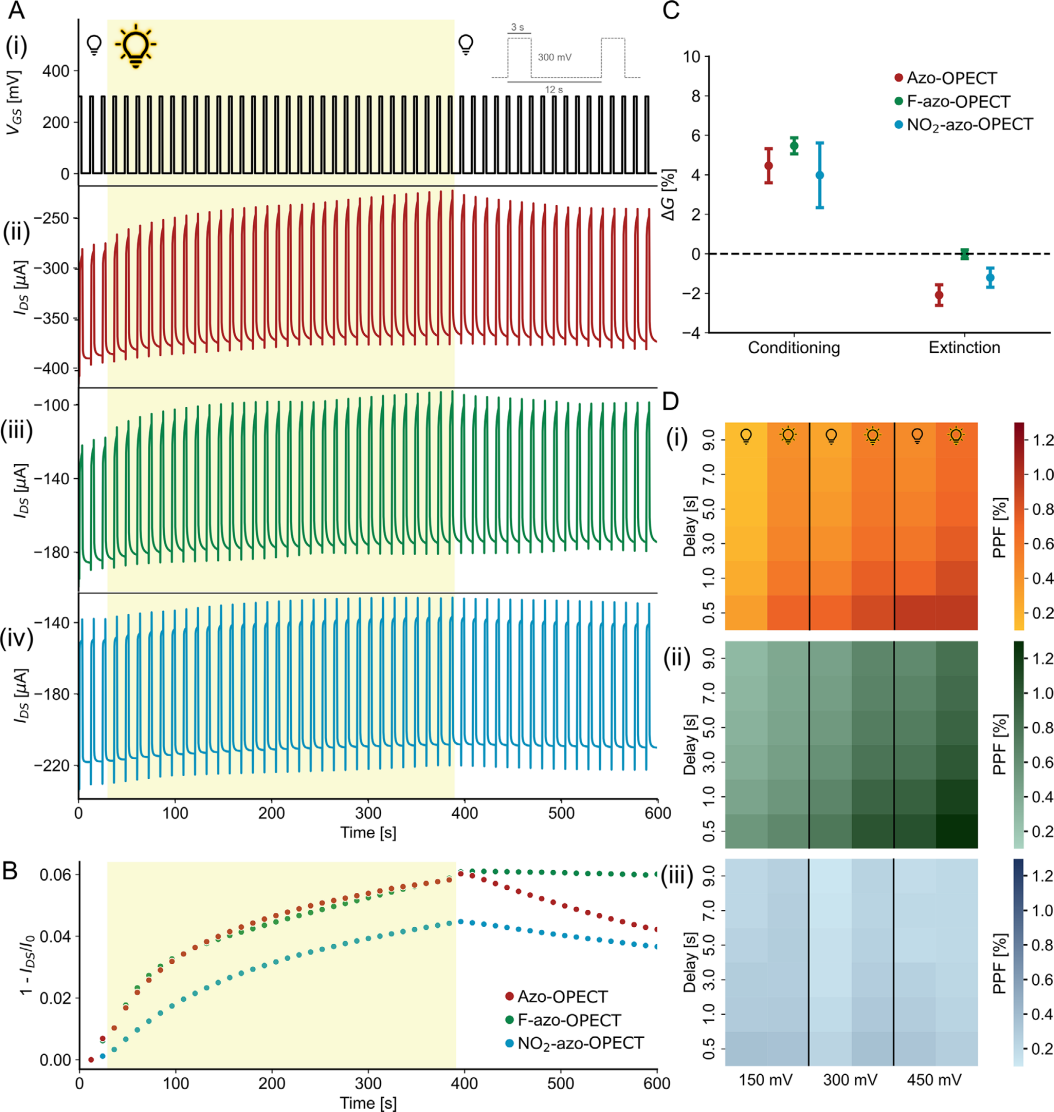
Synaptic conditioning and paired‐pulse facilitation in OPECTs. A) Electrical stimulation at the gate (300 mV, 12 s period, 25% duty cycle) under continuous light illumination (2.96 mW cm^−2^, 365 nm; indicated by yellow background) for 360 s (i), and corresponding channel current responses for azo‐OPECT (ii), F‐azo‐OPECT (iii), and NO_2_‐azo‐OPECT (iv). B) Channel current measured 8 s after each pulse, with baseline offset removed for clarity. C) Percentage variation of channel conductance after 6 min of illumination (conditioning) and following 3 min in the dark (extinction). D) Heat maps of the PPF index as a function of pulse delay (y‐axis) and gate voltage amplitude (x‐axis), in dark and after pre‐illumination, for azo (i), F‐azo (ii), and NO_2_‐azo (iii). Darker colors indicate higher PPF values.

To assess long‐term potentiation and memory retention with combined light and electrical stimuli, a constant light intensity (2.96 mW cm^−2^) was applied for 5 min during transient operation, alongside a train of electrical pulses at the gate (V_GS_ = 300 mV, 3 s on and 12 s off), while maintaining a constant drain‐source voltage of −200 mV (**Figure** **4Ai**). During illumination, the channel current failed to return to baseline after each electrical pulse, indicating sustained modulation of channel conductance that persisted even after the light was turned off (Figure 4Aii–iv). Synaptic plasticity was quantified by calculating the percentage change in channel conductance after 5 min of light exposure, followed by its recovery 3 min after light removal. These changes were determined by comparing conductance values before and after the pulse train. All devices demonstrated light‐induced LTP behavior (Figure 4B,C).

Azo‐OPECTs, previously reported to retain memory following optical stimulation,^[^ ^41^ ^]^ exhibited enhanced LTP in this study when electrical gating was applied concurrently. This enhancement is likely due to a prolonged device turn‐off time after gate stimulation. However, the degree of memory extinction (i.e., conductance recovery in the dark) varied among devices. Azo‐ and NO_2_‐azo‐OPECTs showed partial recovery, while F‐azo‐OPECTs exhibited minimal conductance reversal, indicating more persistent channel modulation and a stronger LTP response (Figure 4B). This behavior may be attributed to the higher surface capacitance and increased hydrophilicity of the F‐azo films, which likely promote sustained polarization and prolonged de‐doping/doping cycles in the PEDOT:PSS channel.

Furthermore, in contrast to optoelectronic devices modulated solely at zero gate bias,^[^ ^49^ ^]^ this study also investigated short‐term plasticity (STP) induced by electrical stimulation using paired‐pulse facilitation (PPF) experiments. PPF is a phenomenon observed in biological synapses, where closely spaced consecutive stimuli lead to enhanced synaptic strength, depending on the inter‐pulse interval.^[^ ^50^ ^]^ Here, synaptic weight modulation was evaluated by applying pairs of electrical pulses with varying time delays and analyzing the change in channel conductance before and after each pair, under both illuminated and dark conditions (Figure S14, Supporting Information). Overall, the results showed an increase in synaptic weight under illumination for the tested delay times and devices, consistent with light‐enhanced synaptic conditioning (Figure 4D). The corresponding color maps indicate facilitation for pulse delays up to 3 s. Notably, NO_2_‐azo‐OPECTs did not exhibit significant PPF at the tested delays even upon light illumination, which is consistent with the limited response to light offered by the material. In contrast, azo‐OPECTs and F‐azo‐OPECTs demonstrated stronger facilitation upon the combined application of light and electrical bias, highlighting the role of light on gate polarization. Specifically, F‐azo‐OPECTs achieved the highest PPF values (Figure S14, Supporting Information), thanks to the higher film capacitance allowing for increased polarization retention.^[^ ^41^ ^]^ These results highlight the improved capability of azo‐ and F‐azo‐based devices to emulate biologically relevant synaptic behaviors.

## 3 Conclusion

This work presents a rational strategy for engineering light‐sensitive, polarizable gate electrodes for organic photoelectrochemical transistors (OPECTs) by functionalizing N_3_‐PEDOT:PSS with azobenzene derivatives bearing targeted nitro and fluorine substituents. By systematically tuning the electron‐withdrawing behavior of these groups, we investigate how molecular‐level variations influence the optoelectronic and electrochemical behavior of the devices, with a particular focus on neuromorphic functionality.

Our findings reveal a remarkable correlation between interfacial capacitance and synaptic‐like behavior. F‐azo‐tz‐PEDOT:PSS exhibits the highest capacitance, leading to enhanced photogating, greater modulation of channel current, and analog plasticity under dual optical and electrical stimulation. In contrast, NO_2_‐azo‐tz‐PEDOT:PSS, despite its pronounced light absorption, shows diminished gating efficiency and key neuromorphic features, likely due to unfavorable isomerization dynamics and energy level alignment.

Electrochemical impedance spectroscopy, cyclic voltammetry, and DFT/TD‐DFT calculations highlight how these substituent‐induced changes influence the gate/electrolyte interface. Enhanced capacitance, particularly in the case of fluorinated gates, facilitates gradual conductance modulation, fundamental for mimicking synaptic behaviors such as paired‐pulse facilitation and voltage‐dependent memory effects. This analog response, enabled by access to intermediate conductance states, is a defining requirement for realistic neuromorphic operation.

These results highlight the role of interfacial capacitance not merely as a passive parameter but as a tunable and functional design element in organic neuromorphic electronics. Through rational modulation of dipolar interactions and surface energetics at the molecular level, we enable fine control over signal integration, temporal response, and memory encoding, which are fundamental features of biological synapses.

Altogether, this study defines a structure–capacitance–function framework for OPECTs and introduces a chemically tunable, biocompatible platform for adaptive bioelectronic systems. The design principles established here are transferable across neuromorphic architectures, including OECTs, and open pathways toward light‐responsive, polarizable organic interfaces for intelligent computing and sensing. Beyond neuromorphic applications, the demonstrated photogating functionality supports broader use in bioelectronics and hybrid optical‐electrical systems. Our modular chemical approach, based on targeted substituent design within OMIEC‐based platforms, offers a scalable, low‐power route to multifunctional organic devices for next‐generation adaptive electronics.

## Conflict of Interest

The authors declare no conflict of interest.

## Supporting information



Supporting Information

## Data Availability

The data that support the findings of this study are available from the corresponding author upon reasonable request.
