# Supplementary material for: Designing Light‐Sensitive Organic Semiconductors with Azobenzenes for Photoelectrochemical Transistors as Neuromorphic Platforms
Source: Adv Sci (Weinh). 2025 Jul 29;12(39):e09125. doi: 10.1002/advs.202509125 (PMC12533332; doi:10.1002/advs.202509125)
Supplement: Supplementary file 1 — Supporting Information [file ADVS-12-e09125-s001.docx]

Supporting Information

**Designing Light-sensitive Organic Semiconductors with Azobenzenes for Photoelectrochemical Transistors as Neuromorphic Platforms**

*Isabela Berndt Paro,^#^ Martina Gini,^#^ Francesca D’ Elia,^#^ Arianna Massaro, Federica Corrado, Daniela Rana, Ana Varela, Giulia Elli, Matthias Baumann, GiovanniMaria Piccini, Luisa Petti, Daniele Leonori, Ana Belen Muñoz-García, Michele Pavone, Andreas Offenhäusser, Valeria Criscuolo,* Francesca Santoro**

**Experimental Section**

*Materials:* All materials were used as received. 4-(phenylazo)phenol, 4-(4-nitrophenylazo)phenol, sodium azide (NaN_3_), propargylbromide, sodium L-ascorbate, and 3-Glycidyloxypropyltrimethoxysilane (GOPS) were purchased by TCI Deutschland GmbH (Germany). 2-Chloromethyl-2,3-dihydrothieno[3,4-b]-1,4-dioxine (EDOT-Cl), Poly(sodium-4-styrene sulfonate) (PSSNa, Mw 1,000,000), 3-fluoroaniline, potassium carbonate (K_2_CO_3_), sodium sulfate (Na_2_SO_4_), sodium nitrite (NaNO_2_), ammonium chloride (NH_4_Cl), ethyl acetate (EtOAc), acetone, isopropanol (IPA), tetrahydrofuran (THF), acetonitrile (ACN), petroleum ether (PE), dichloromethane (CH_2_Cl_2_), pentane, Alconox soap, ethylene glycol, and 4-Dodecylbenzenesulfonic acid (DBSA) were purchased by Sigma Aldrich Chemie GmbH (Germany). Copper(II) sulphate pentahydrate (CuSO_4_.5H_2_O) and N,N-dimethylformamide (DMF) were purchased by VWR International GmbH (Germany). Dulbecco’s phosphate buffer (DPBS, no calcium, no magnesium) was purchased by Life Technologies GmbH (Germany). Patterned ITO-coated glasses were purchased by Kintec Company (Hong Kong). Platinum-coated titanium mesh and Ag/AgCl 3M KCl electrodes were purchased by Redox.me (Sweden). Dowsil^TM^ 184 Silicone Elastomer (Sylgard 184) was purchased from Farnell (Germany). Clevios PH1000 was purchased from Heraeus Deutschland GmbH & Co. KG (Germany). Kapton tape Tesa 51408 was purchased by RS Components GmbH (Germany).

*Synthesis of 2-Azidomethyl-2,3-dihydrothieno[3,4-b]-1,4-dioxine (EDOT-N_3_):* EDOT-N_3_ was synthesized as previously reported with slight modifications.^[1]^ In brief, 2-chloromethyl-2,3-dihydrothieno[3,4-b]-1,4-dioxine (EDOT-Cl) (200 mg, 1 mmol, 1 eq) was dissolved in DMF (12 mL) and stirred at r.t. under inert atmosphere until complete dissolution of the product, followed by the addition of NaN_3_ (136 mg, 2 mmol, 2 eq) and the reaction mixture was refluxed for 3 hours. DMF was removed under reduced pressure, and the crude mixture was redissolved and extracted with EtOAc and H_2_O. The organic layer was dried over anhydrous Na_2_SO_4_, filtered and evaporated under reduced pressure yielding EDOT-N_3_ as a colorless oil (196.5 mg, 0.99 mmol, 99%). TLC PE/EtOAc 10:1. ^1^H NMR (CDCl_3_, 400 MHz): 3.56 (2H, ddd), 4.07 (1H, dd), 4.21 (1H, dd), 4.35 (1H, m), 6.40 (2H, dd).

*Synthesis of 4-Propargyloxyazobenzene (azoalkyne):* Azoalkyne was synthesized as previously reported.^[1]^ In brief, 4-(Phenylazo)phenol (400 mg, 2 mmol, 1 eq) was dissolved in previously degassed acetone (24 mL). K_2_CO_3_ (1.38 g, 10 mmol, 5 eq) was added to the organic solution and the reaction mixture was stirred at r.t. under inert atmosphere for 90 minutes. Then, propargyl bromide (1.19 mg, 10 mmol, 5 eq) was added, and the reaction mixture was refluxed overnight, under inert atmosphere. Acetone was removed under reduced pressure and the crude mixture was redissolved and extracted with EtOAc and H_2_O. The organic layer was dried over anhydrous Na_2_SO_4_, filtered and evaporated under reduced pressure yielding the azo-alkyne as an orange powder (465 mg, *1.97* mmol, 98.5%). TLC PE/ EtOAc 10:1. ^1^H NMR (CDCl_3_, 400 MHz): δ 7.94 (2H, m), 7.89 (2H, dt), 7.54–7.42 (3H, m), 7.12 (2H, m), 4.78 (2H, d), 2.57 (t, 1H).

*Synthesis of (4-nitrophenyl)-2-(4-(prop-2-yn-1-yloxy) phenyl) diazene (NO_2_-azoalkyne)*: NO_2_-azoalkyne was synthesized adapting the previously reported procedure.^[1]^ 4-(4-nitrophenylazo)phenol (NO_2_-azo-OH) (200 mg, 0.82 mmol, 1 eq) was dissolved in ACN (20 mL) at 80°C until complete dissolution. K_2_CO_3_ (98 mg, 2.79 mmol, 3.4 eq) was added to the hot organic solution which turned dark red in color. Then, propargyl bromide (120 mg, 1 mmol mmol, 1.24 eq) was added, and the reaction mixture was refluxed overnight. ACN was removed under reduced pressure and the crude mixture was redissolved and extracted with EtOAc and H_2_O. The organic layer was dried over anhydrous Na_2_SO_4_, filtered and evaporated under reduced pressure to yield NO_2_-azoalkyne as an orange powder (189.7 mg, 0.675 mmol, 82.3%) TLC PE/ EtOAc 10:1. ^1^H NMR (600 MHz, DMSO-d_6_): δ 8.43 (2H, d, *J* 8.4 Hz), 8.05 (2H, d, *J* 12 Hz), 8.00 (2H, d, *J* 12 Hz), 7.24 (2H, d, *J* 12 Hz), 4.98 (2H, s), 3.67 (1H, s). ^13^C NMR (151 MHz, DMSO-d_6_): δ 161.43, 155.82, 147.12, 125.76, 125.55, 123.69, 116.21, 79.34, 79.12, 56.48.

*Synthesis of 1-(3-Fluorophenyl)-2-(4-(prop-2-yn-1-yloxy)phenyl)diazene (F-azoalkyne)*: F-azoalkyne was synthesized in two steps.

*4-((3-Fluorophenyl)diazenyl)phenol (F-azophenol).*^[2]^ 3-fluoroaniline (2 g, 18 mmol) was dissolved in 6 M HCl (5 mL) and the solution was cooled to 0 °C. An aqueous solution of NaNO_2_ (1.37 g, 19.8 mmol), precooled to 0 °C, was added dropwise to the reaction mixture under stirring and the mixture was kept stirring at 0 °C for 10 minutes. A precooled solution of the corresponding phenol (1.69 g, 18 mmol)) in 2 M NaOH (7.5 mL) was added dropwise and the mixture was stirred at 0 °C for 30 min. The resulting precipitate was filtered and washed with water (20 mL). EtOAc (50 mL) was added, and the aqueous layer was extracted with EtOAc. The organic layers were washed with Brine, dried over Na_2_SO_4_ and concentrated under reduced pressure, then combined with the precipitate and purified by column chromatography on silica gel chromatography (eluant: CH_2_Cl_2_), to yield F-azophenol as an orange solid (467 mg, 12%). ^1^H NMR (600 MHz, CDCl_3_): δ 7.89 (2H, d, *J* 9.0 Hz), 7.71 (1H, ddd, *J* 8.0, 2.0, 1.0 Hz), 7.56 (1H, dt, *J* 10.0, 2.0 Hz), 7.47 (1H, td, *J* 8.0, 5.9 Hz), 7.15 (1H, dd, *J* 8.2, 2.6, 1.0 Hz), 6.95 (2H, d, *J* 9.0 Hz), 5.14 (1H, s). ^19^F NMR (564 MHz, CDCl_3_): δ –112.27 (td, *J* 9.0, 6.0 Hz). ^13^C NMR (150 MHz, CDCl_3_): δ 163.3 (d, *J_C-F_* 247 Hz), 158.6, 154.2 (d, *J_C-F_* 6.7 Hz), 147.0, 130.2 (d, *J_C-F_* 8.7 Hz), 125.3, 120.1 (d, *J_C-F_* 2.5 Hz), 117.1 (d, *J_C-F_* 21.8 Hz), 115.9, 107.8 (d, *J_C-F_* 23.7 Hz).

*1-(3-Fluorophenyl)-2-(4-(prop-2-yn-1-yloxy)phenyl)diazene:* The F-azophenol (467 mg, 2.16 mmol) was dissolved in DMF (4.3 mL). K_2_CO_3_ (891 mg, 6.45 mmol) and propargyl bromide (767 mg, 6.45 mmol) were added, the mixture was heated to 70 °C and stirred for 16 h. Saturated aqueous NH_4_Cl (50 mL) and EtOAc (50 mL) were added and the layers were separated. The aqueous layer was extracted with EtOAc and the combined organic layers were washed with Brine, dried over Na_2_SO_4_ and concentrated under reduced pressure. The crude product was purified by column chromatography on silica gel (eluant: pentane/EtOAc 95:5) to yield F-azoalkyne as an orange solid (521 mg, 95%). ^1^H NMR (600 MHz, CDCl_3_): δ 7.94 (2H, d, *J* 9.2 Hz), 7.72 (1H, d, *J* 7.6 Hz), 7.57 (1H, dt, *J* 9.4, 2.1 Hz), 7.47 (1H, td, *J* 8.1, 6.0 Hz), 7.15 (1H, td, *J* 8.1, 2.1 Hz), 7.11 (2H, d, *J* 9.2 Hz), 4.79 (2H, d, *J* 2.3 Hz), 2.57 (1H, t, *J* 2.3 Hz). ^19^F NMR (564 MHz, CDCl_3_): δ –112.24 (m). ^13^C NMR (150 MHz, CDCl_3_): δ 163.4 (d, *J_C-F_* 247 Hz), 160.2, 154.2 (d, *J_C-F_* 6.9 Hz), 147.3, 130.2 (d, *J_C-F_* 8.6 Hz), 124.9, 120.2 (d, *J_C-F_* 3.0 Hz), 117.2 (d, *J_C-F_* 22 Hz), 115.2, 107.9 (d, *J_C-F_* 23 Hz), 78.0, 76.1, 56.0.

*Device fabrication*. Patterned indium tin oxide (ITO)-coated glass slides (25 mm × 25 mm) with a three-pad configuration (**Figure 1A**) were used as substrates. Prior to use, substrates were cleaned in an aqueous Alconox solution (10 g L^-1^), followed by sequential sonication in deionized water, acetone, and isopropanol for 10 minutes at high power.

OPECT gate electrodes were fabricated by functionalizing electrodeposited N_3_-PEDOT:PSS films with azoalkyne derivatives *via* click chemistry. The synthesized EDOT-N_3_ monomer was dissolved in deionized water (0.01 M) containing 7.5 mg mL^-1^ of PSSNa. Prior to electrodeposition, the dispersion was vortexed manually for 20 seconds, sonicated for 10 minutes at full power, and stirred for 30 minutes. Electrodeposition was performed using a three-electrode setup: a platinum-coated titanium mesh (25 mm × 25 mm) as the counter electrode (CE), the cleaned ITO substrate as the working electrode (WE), and an Ag/AgCl (3 M KCl) reference electrode (RE). N_3_-PEDOT:PSS films were deposited by CV using a VSP-300 potentiostat/galvanostat (Bio-Logic Science Instruments GmbH, Germany), sweeping the potential from 0 V to 1.15 V over 10 cycles at a scan rate of 50 mV s^-1^, resulting in a film thickness of 120.69 ± 4.42 nm. After deposition, the films were rinsed with deionized water, dried with nitrogen, and annealed on a hotplate at 120 °C for 1 hour.

The resulting films were then functionalized *via* copper(I)-catalyzed azide–alkyne cycloaddition (CuAAC). The appropriate alkyne-functionalized azobenzene derivative (47 mg azoalkyne, 56 mg NO_2_-azoalkyne, or 50.6 mg F-azoalkyne; 0.6 mmol) was dissolved in 10 mL of THF. An aqueous solution (10 mL) containing CuSO_4_·5H_2_O (50 mg, 0.64 mmol, 0.89 eq.) and sodium L-ascorbate (39.6 mg, 0.53 mmol, 0.89 eq.) was added. The reaction mixture was transferred into a custom-designed polyetheretherketone (PEEK) chamber equipped with a stirring mechanism to ensure constant agitation in a sealed environment. The N_3_-PEDOT:PSS films were fully submerged in the mixture and stirred at 400 rpm for 24 hours. After functionalization, the films (azo-tz-PEDOT:PSS, NO_2_-azo-tz-PEDOT:PSS, or F-azo-tz-PEDOT:PSS) were thoroughly rinsed with THF and deionized water, dried under nitrogen, and characterized (thicknesses: azo-tz-PEDOT:PSS, 186.28 ± 5.88 nm; NO_2_-azo-tz-PEDOT:PSS, 167.32 ± 11.15 nm; F-azo-tz-PEDOT:PSS, 183 ± 5.87 nm).

The OECT channel was fabricated by spin coating a PEDOT:PSS blend using a Kapton tape hard mask. The gate area was first protected with a PDMS mask (13 mm × 25 mm × 1 mm), prepared by mixing Sylgard 184 base and curing agent (10:1 ratio) and cured at 120 °C for 1 hour. A 12 mm × 25 mm strip of Kapton tape was adhered to the remaining area, and a central opening (7 mm × 15 mm) was cut to expose the substrate. The PEDOT:PSS blend (94 vol% Clevios PH1000, 5 vol% ethylene glycol, 1 vol% GOPS, and 0.02 vol% DBSA) was prepared and sonicated at full power for 30 minutes. After oxygen plasma activation (50 W, 80 sccm, 2 minutes), the blend was spin coated at 2000 rpm for 2 minutes, producing a film with a thickness of 103.0 ± 7.1 nm. The PDMS mask was then carefully removed, and the sample was annealed at 140 °C for 1 hour before removing the Kapton tape.

*Fourier Transform Infrared spectroscopy.* The infrared spectra were obtained using an FTIR spectrometer Invenio R (Bruker). Data was collected from wavelength 4000 to 400 cm^-1^, with a resolution of 4 cm^-1^; 64 scans were performed with a total measurement time of 35 seconds.

*Optical characterization.* Optical transmittance spectra were measured by using a Lambda 900 spectrophotometer (Perkin Elmer). The measurements were performed on solutions (10^-5^ M in THF) and films in dark conditions (*i.e.,* no UV illumination). The films were then exposed to UV light illumination (wavelength of 365 nm, intensity of 0.31 mW cm^-2^) for different time intervals. The evolution of the absorbance at 365 nm showed an exponentially decaying behavior that was fitted to extract the time constant of the *trans-*to*-cis* isomerization.

*Atomic Force Microscopy*. A Dimension Icon® AFM (Bruker Corporation, USA) equipped with ScanAsyst-fluid cantilever (Brucker, USA) under ScanAsyst mode was used in dry conditions and under ambient atmosphere to measure films surface topography and roughness. An area of 5 × 5 µm^2^ was scanned operating in tapping mode with a scan rate of 2 Hz. Nanoscope 2.0 software was used to evaluate the root mean square (RMS or Rq) roughness for each acquired area.

*Contact angle.* OCA 20 (DataPhysics Instruments, GmbH), was used to determine the contact angle of the pristine films by using the static sessile drop method. 20 μL of Milli-Q water was disposed on the surface of the films and a photograph of the droplet was taken. After setting the baseline, the profile of the droplet was fitted to extract the contact angle.

*Cyclic voltammetry.* CV measurements were performed using VSP-300 potentiostat/galvanostat (Bio-Logic Science Instruments GmbH, Germany), using DPBS (no calcium, no magnesium) as electrolyte (0.7 mL, confined in a well of diameter = 1 cm). A platinum wire was used as CE, the aqueous Ag/AgCl 3M KCl as RE, while the portion of ITO substrate not covered by the polymeric film as WE. For stabilization of the polymeric films, the samples were immersed in DPBS for one hour prior measurement. A voltage sweep in the range of [-0.6, 0.6 V] was applied for 3 cycles at 50 mV s^-1^ scan rate. The measurements were performed in dark conditions (*i.e.,* no UV illumination), and after 1 and 6 minutes of illumination (wavelength of 365 nm, intensity of 0.92, 2.96 and 4.73 mW cm^-2^).

*Electrochemical impedance spectroscopy.* EIS was performed using VSP-300 potentiostat/galvanostat (Bio-Logic Science Instruments GmbH, Germany) on the same set up described for CV above, in a dedicated well positioned on a portion of pristine film. In this case, an Ag/AgCl pellet electrode was used as RE. The samples were immersed in DPBS for one hour prior measurement. Frequencies from 10 kHz to 0.1 Hz were scanned, using AC voltages with an amplitude of 10 mV. The measurements were performed in dark conditions (*i.e.,* no UV illumination), and after 1 and 6 minutes of illumination (wavelength of 365 nm, intensity of 0.92, 2.96 and 4.73 mW cm^-2^).

*Electrical characterization of OPECTs*. All measurements were performed following an initial swelling step, where the films were immersed in deionized water for 24 hours. A PDMS mold was used to confine the DPBS electrolyte (150 µL), defining a gate active area of 4 mm × 7 mm, a channel active area of 4 mm × 7 mm, and a gate-to-channel distance of 2 mm. Measurements were carried out using the Arkeo measurement system (Cicci Research, Italy). The system’s probes, used to contact the electrodes, integrate force and sensing by short-circuiting the terminals. This configuration enables a closed-loop feedback mechanism that continuously monitors and adjusts the applied voltage to ensure accurate delivery of the programmed values.

*Steady state measurements.* Output and transfer characteristics were recorded by sweeping the gate voltage (V_GS_ from –0.2 to 0.8 V, scan rate of 200 mV s^-1^) and the drain voltage (V_DS_ from –0.6 to 0.1 V), respectively. Measurements were performed under dark conditions and following 2 minutes and 5 minutes of UV illumination (365 nm) at different light intensities (0.92, 2.96, and 4.73 mW cm^-2^, corresponding to 20%, 60%, and 100% of the source power). Between measurements, the devices were thermally treated on a hotplate at 80 °C for 15 minutes to restore the initial state.

*Transient measurements: light stimulation at zero gate voltage bias.* Fixed voltages V_DS_ = -200 mV and V_GS_ = 0 mV were applied. After a 60-second baseline period, the gate electrode was exposed to a train of light pulses (365 nm, 2.96 mW cm^-2^), and both the photocurrent generated at the gate and the channel current were recorded. Two light stimulation protocols were used: a slow stimulation regime with a 120-second period and 50% duty cycle, and a fast stimulation regime with a 6-second period and 50% duty cycle. Both protocols provided the same total illumination time of 180 seconds. Devices were thermally reset between experiments.

*Transient measurements: electrical stimulation in dark and light conditions.* A train of electrical pulses was applied at the gate electrode (300 mV, 12 s period, 25% of duty cycle, 50 cycles), while keeping the drain voltage fixed at V_DS_ = -200 mV. Both gate and channel currents were recorded. The experiment was repeated under illumination (365 nm, 2.96 mW cm^-2^), following a sequence of 30 seconds in the dark, 360 seconds under illumination, and 210 seconds in the dark.

*Transient measurements: Paired Pulsed Facilitation in dark and light conditions.* To evaluate PPF, pairs of electrical pulses with varying inter-pulse delays (Δt = 0.5, 1, 3, 5, 7, and 9 s), spaced 20 seconds apart, were applied to the gate electrode. This was performed at three different voltage amplitudes (V_GS_ = 150 mV, 300 mV, 450 mV), while maintaining a constant drain at V_DS_ = -200 mV. The resulting gate and channel currents were recorded. Measurements were conducted under dark conditions and again after 5 minutes of illumination at 365 nm with a light intensity of 2.96 mW cm^-2^.

*Computational details.* The photophysical properties of F-azo-tz-PEDOT and NO_2_-azo-tz-PEDOT were investigated via state-of-the-art density functional theory (DFT) calculations with the Gaussian16 suite of Quantum Chemistry programs.^[3]^ For all minimum-energy structural optimizations and ground-state electronic properties, we applied a standard level of theory with the reliable B3LYP hybrid density functional^[4]^ and Pople’s double-z basis sets 6-31 G(d,p) for all atoms.^[5,6]^ Vertical electronic excitation and excited-state properties of *cis* and *trans* F-azo-tz-PEDOT and NO_2_-azo-tz-PEDOT systems were characterized with time-dependent DFT (TD-DFT) calculations at the same level of theory.^[7]^

*Data analysis*. All experiments were performed in triplicate. The reported data represent the average of three independent measurements, while the graphs display representative results.

Unless otherwise noted, all calculations were performed using custom Python scripts. Numerical fitting of the EIS data was carried out using DearEIS, a Python package for impedance spectroscopy analysis. The fitting procedure involved designing the equivalent circuit model and applying a least-squares optimization function (NumPy package).

Injected charge was calculated by integrating the area under the gate current curve over 60 seconds of illumination. The variation in channel conductance after light stimulation was determined by comparing the current measured before illumination (at 59 s) and after the full illumination protocol (360 s for slow pulses, 417 s for fast pulses).

For the electrical pulsing experiments, baseline channel currents were extracted 8 seconds after the end of each pulse. Synaptic conditioning behavior was quantified using baseline currents from pulse 3 (dark condition) and pulse 33 (light condition), while extinction was evaluated by comparing baseline values between pulse 33 (light condition) and pulse 49 (dark condition).

PPF was calculated by measuring the change in conductance between the baseline current before the first pulse and after the second pulse in each pair, thus quantifying the effect of two consecutive stimuli on synaptic-like response.

References

[1] F. Corrado, U. Bruno, M. Prato, A. Carella, V. Criscuolo, A. Massaro, M. Pavone, A. B. Muñoz-García, S. Forti, C. Coletti, O. Bettucci, F. Santoro, *Nat Commun* 2023, *14*, 6760.

[2] M. R. Yazdanbakhsh, M. Giahi, A. Mohammadi, *Journal of Molecular Liquids* **2009**, *144*, 145.

[3] Gaussian 16, Revision B.01, Frisch, M.J., Trucks, G.W., Schlegel, H.B., Scuseria, G.E., Robb, M.A., Cheeseman, J.R.; Scalmani, G.; Barone, V.; Petersson, G.A.; Nakatsuji, H.; Li, X.; Caricato, M.; Marenich, A.V.; Bloino, J., Janesko, B.G., Gomperts, R., Mennucci, B., Hratchian, H.P., Ortiz, J.V., Izmaylov, A.F., Sonnenberg, J.L., Williams-Young, D., Ding, F., Lipparini, F., Egidi, F., Goings, J., Peng, B., Petrone, A., Henderson, T., Ranasinghe, D., Zakrzewski, V.G., Gao, J., Rega, N., Zheng, G., Liang, W., Hada, M., Ehara, M., Toyota, K., Fukuda, R., Hasegawa, J., Ishida, M., Nakajima, T., Honda, Y., Kitao, O., Nakai, H., Vreven, T., Throssell, K., Montgomery Jr., J.A., Peralta, J.E., Ogliaro, F., Bearpark, M.J., Heyd, J.J., Brothers, E.N., Kudin, K.N., Staroverov, V.N., Keith, T.A., Kobayashi, R., Normand, J., Raghavachari, K., Rendell, A.P., Burant, J.C., Iyengar, S.S., Tomasi, J., Cossi, M., Millam, J.M., Klene, M., Adamo, C., Cammi, R., Ochterski, J.W., Martin, R.L., Morokuma, K., Farkas, O., Foresman, J.B., Fox, D.J. Gaussian, Inc., Wallingford CT (2016) GaussView 5.0. Wallingford, E.U.A. - References - Scientific Research Publishing, **2022**.

[4] A. D. Becke, *The Journal of Chemical Physics* **1993**, *98*, 5648.

[5] V. A. Rassolov, M. A. Ratner, J. A. Pople, P. C. Redfern, L. A. Curtiss, *Journal of Computational Chemistry* **2001**, *22*, 976.

[6] M. M. Francl, W. J. Pietro, W. J. Hehre, J. S. Binkley, M. S. Gordon, D. J. DeFrees, J. A. Pople, *The Journal of Chemical Physics* **1982**, *77*, 3654.

[7] A Chemist’s Guide to Density Functional Theory, 2nd Edition | Wiley

**Supplementary Information Section**


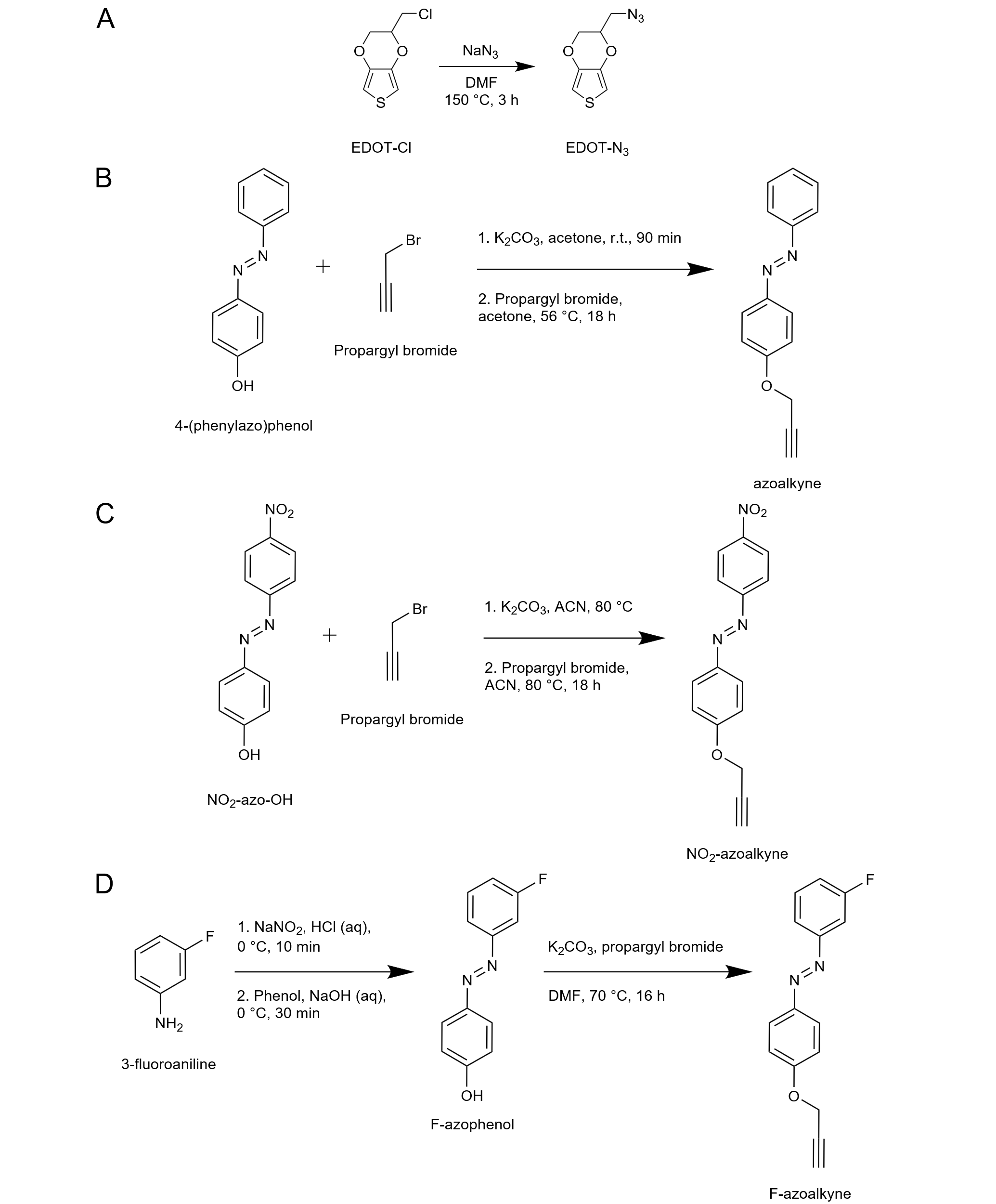


**Supplementary Figure S1. Synthetic procedure.** A) Synthesis of EDOT-N_3_, B) synthesis of azoalkyne, C) synthesis of NO_2_-azoalkyne and D) synthesis of F-azoalkyne. Detailed protocols are reported in the Experimental Section.


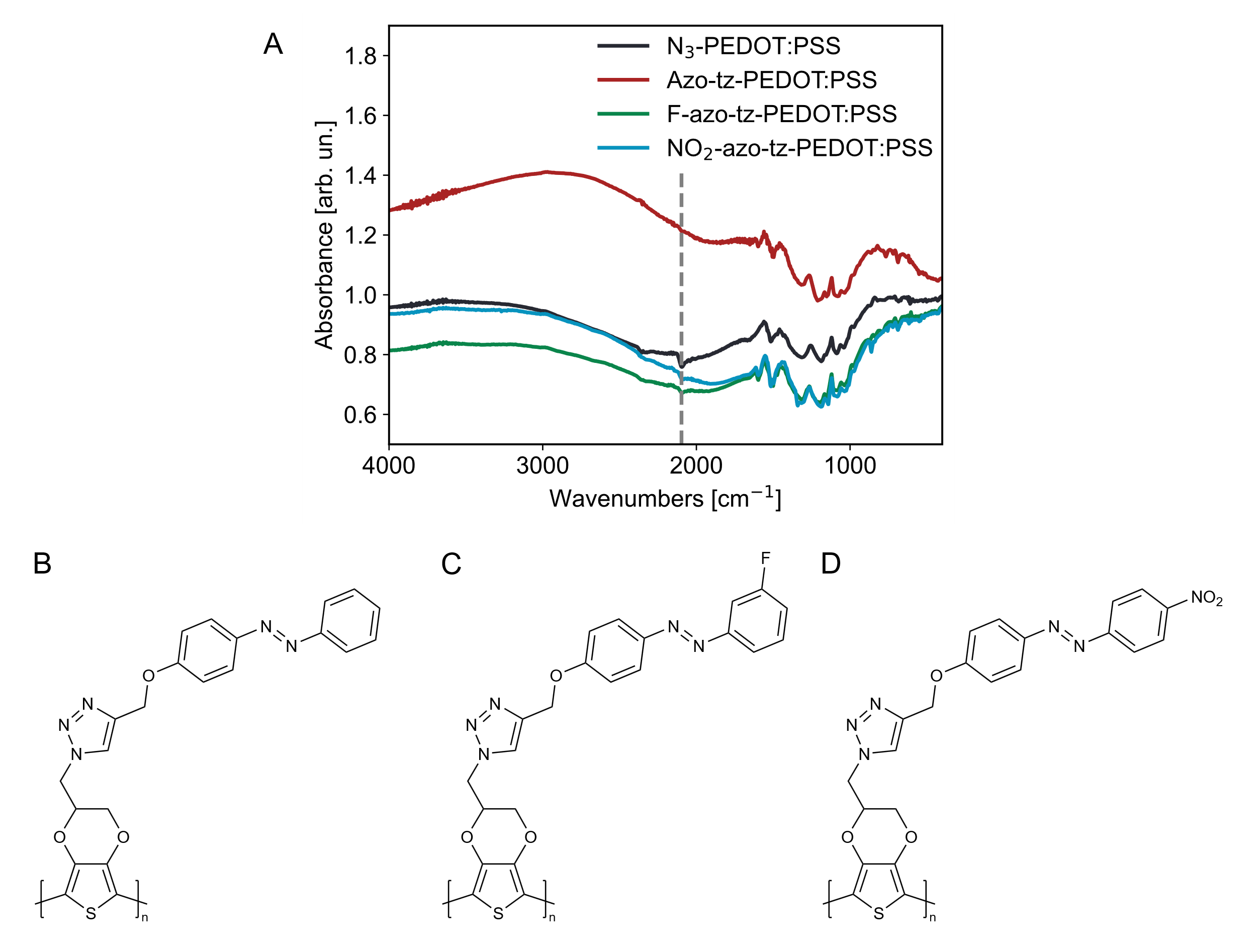


**Supplementary Figure S2.** **FT-IR spectra and molecular structures of X-azo-tz-PEDOT:PSS films.**
A) FTIR-ATR spectra of N₃-PEDOT:PSS (black line), azo-tz-PEDOT:PSS (red line), F-azo-tz-PEDOT:PSS (green line), and NO_2_-azo-tz-PEDOT:PSS (blue line). A decrease in the azide stretching vibration band at 2097 cm^-1^ is observed upon functionalization, confirming the successful conversion of the azide group into the triazole ring *via* click chemistry. B–D) Extended chemical structures of the functionalized polymers: B) azo-tz-PEDOT, C) F-azo-tz-PEDOT, and D) NO_2_-azo-tz-PEDOT.


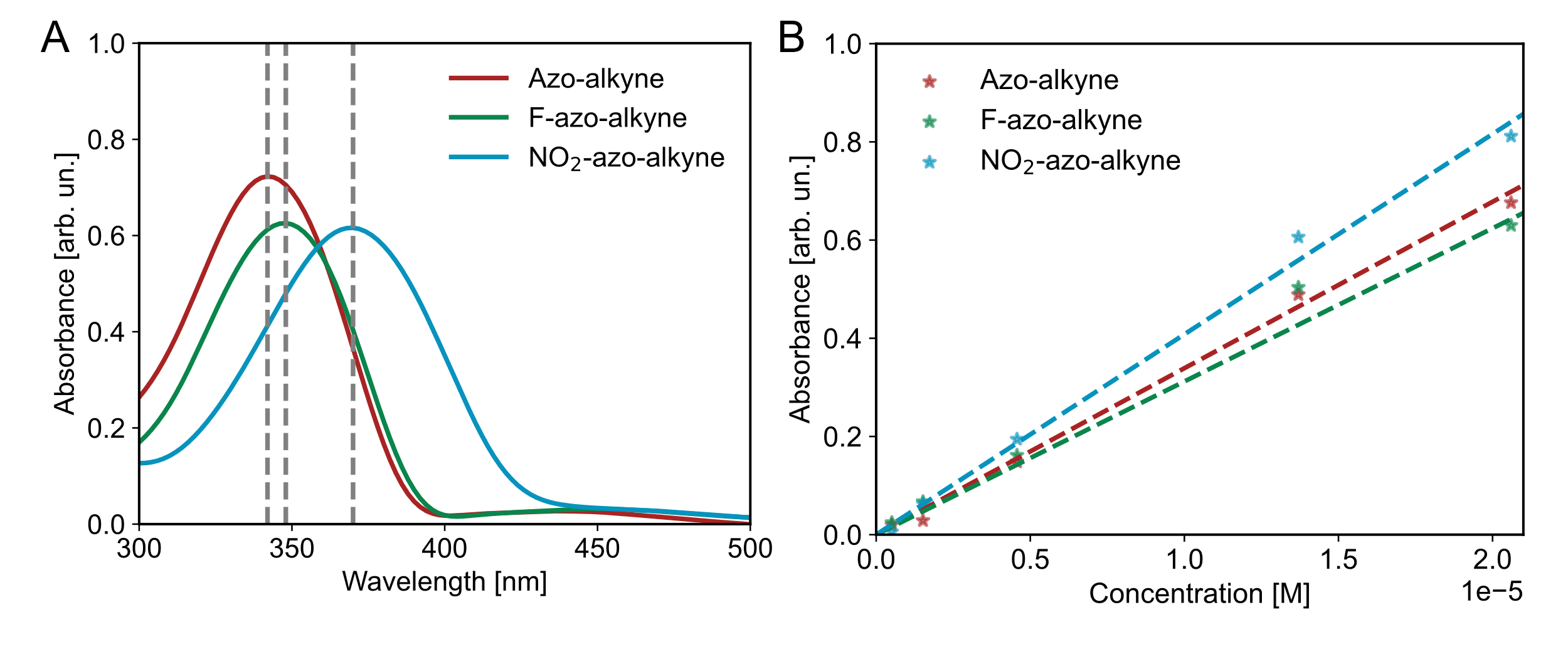


**Supplementary Figure S3. Absorbance spectra of X-azoalkynes.** A) UV-vis spectra of azoalkyne (red line), F-azoalkyne (green line), and NO_2_-azoalkyne (blue line), recorded in THF solution at concentration 10^-5^ M. The dashed grey line highlights the absorption maxima of the three compounds: azoalkyne λ_max_ = 342 nm; F-azoalkyne λ_max_ = 348 nm; NO_2_-azoalkyne λ_max_  = 370 nm. B) Molar extinction coefficient (ε) calculated for each compound: azoalkyne 33.9 ± 1.3 × 10^3^ M^-1^ cm^-1^, F-azoalkyne 31.2 ± 2.2 × 10^3^ M^-1^ cm^-1^, and NO_2_-azoalkyne 40.8 ± 1.9 × 10^3^ M^-1^ cm^-1^.


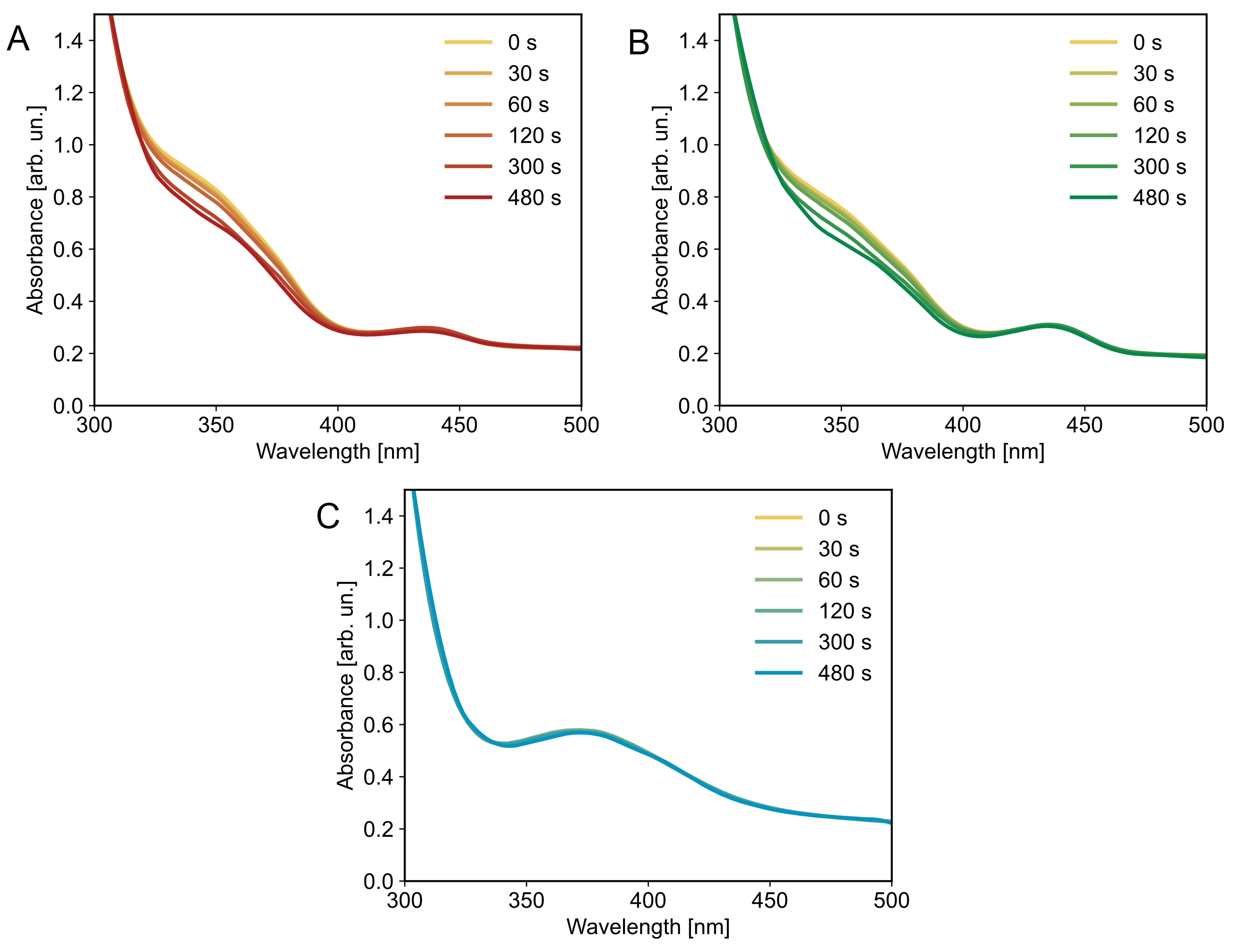


**Supplementary Figure S4. Time-dependent optical switching.** UV-vis spectra of A) azo-tz-PEDOT:PSS, B) F-azo-tz-PEDOT:PSS, and C) NO_2_-azo-tz-PEDOT:PSS films recorded over time under increasing UV illumination (λ = 365 nm, intensity = 0.31 mW cm^-2^).


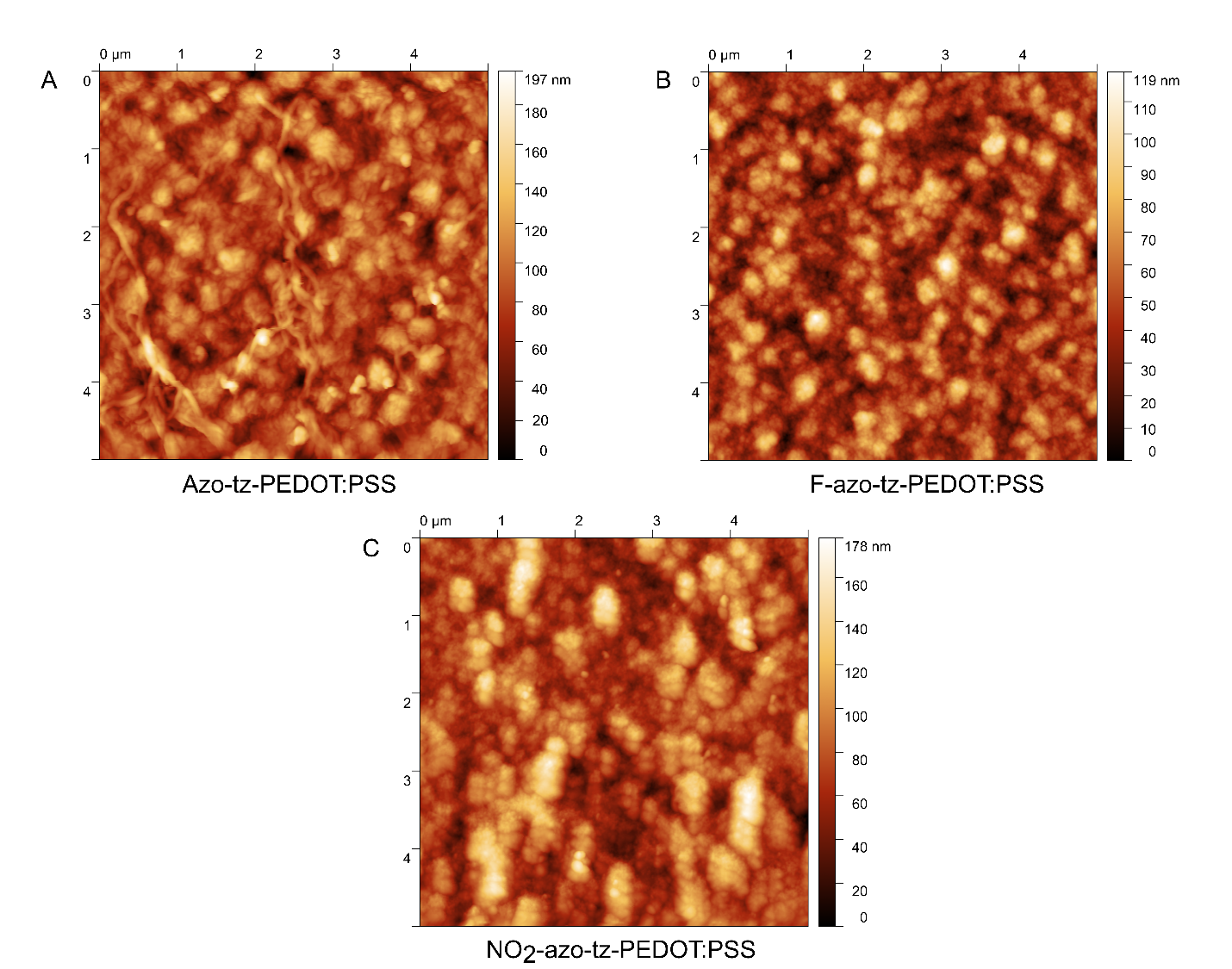


**Supplementary Figure S5. Atomic force microscopy of gate electrodes.** Atomic force micrographs of A) azo-tz-PEDOT:PSS, B) F-azo-tz-PEDOT:PSS, and C) NO_2_-azo-tz-PEDOT:PSS films. The analysis revealed no significant differences in surface topography among the films. The mean RMS roughness values were 31.8 ± 2.8 nm for azo-tz-PEDOT:PSS, 16.1 ± 1.2 nm for F-azo-tz-PEDOT:PSS, and 27.2 ± 0.9 nm for NO_2_-azo-tz-PEDOT:PSS (N = 3).

| **Film** | **ϴ_c_ (°)** |
| --- | --- |
| Azo-tz-PEDOT:PSS | 98.0 ± 1.0 |
| F-azo-tz-PEDOT:PSS | 57.9 ± 0.9 |
| NO_2_-azo-tz-PEDOT:PSS | 66.2 ± 3.4 |

**Supplementary Table S1**. **Contact angle measurements**. Surface hydrophilicity was assessed through contact angle measurements, revealing that azo-tz-PEDOT:PSS is more hydrophobic compared to the other films (N = 3).


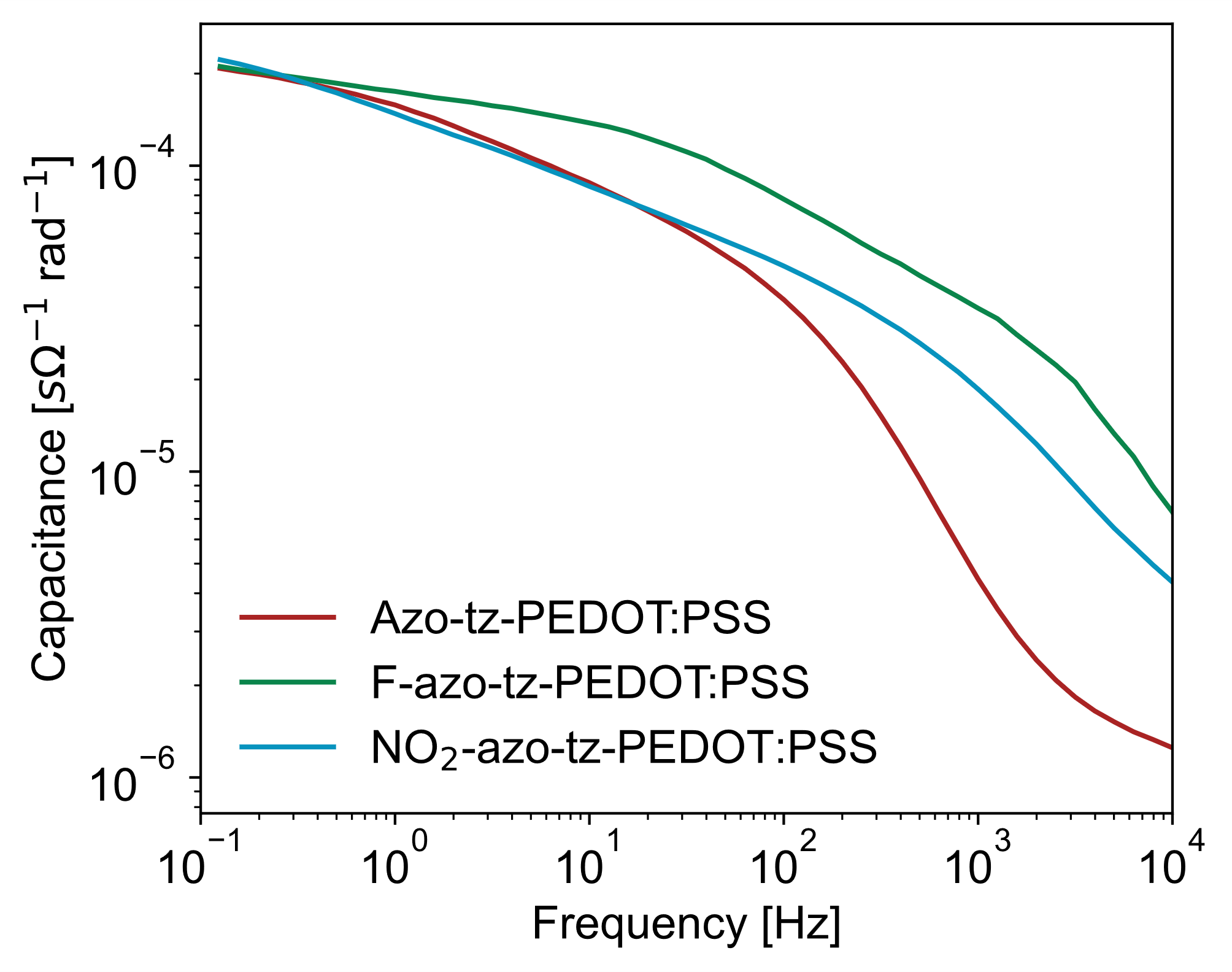


**Supplementary Figure S6. Capacitance of the films measured by EIS under dark conditions.** Capacitance of azo- (red line), F-azo- (green line), and NO_2_-azo-tz-PEDOT:PSS (blue line) films calculated from EIS spectra as $C = -\frac{1}{2\cdot\pi\cdot f\cdot Z_{im}}$, where *f* is the frequency and *Z*_im_ is the imaginary part of the impedance.


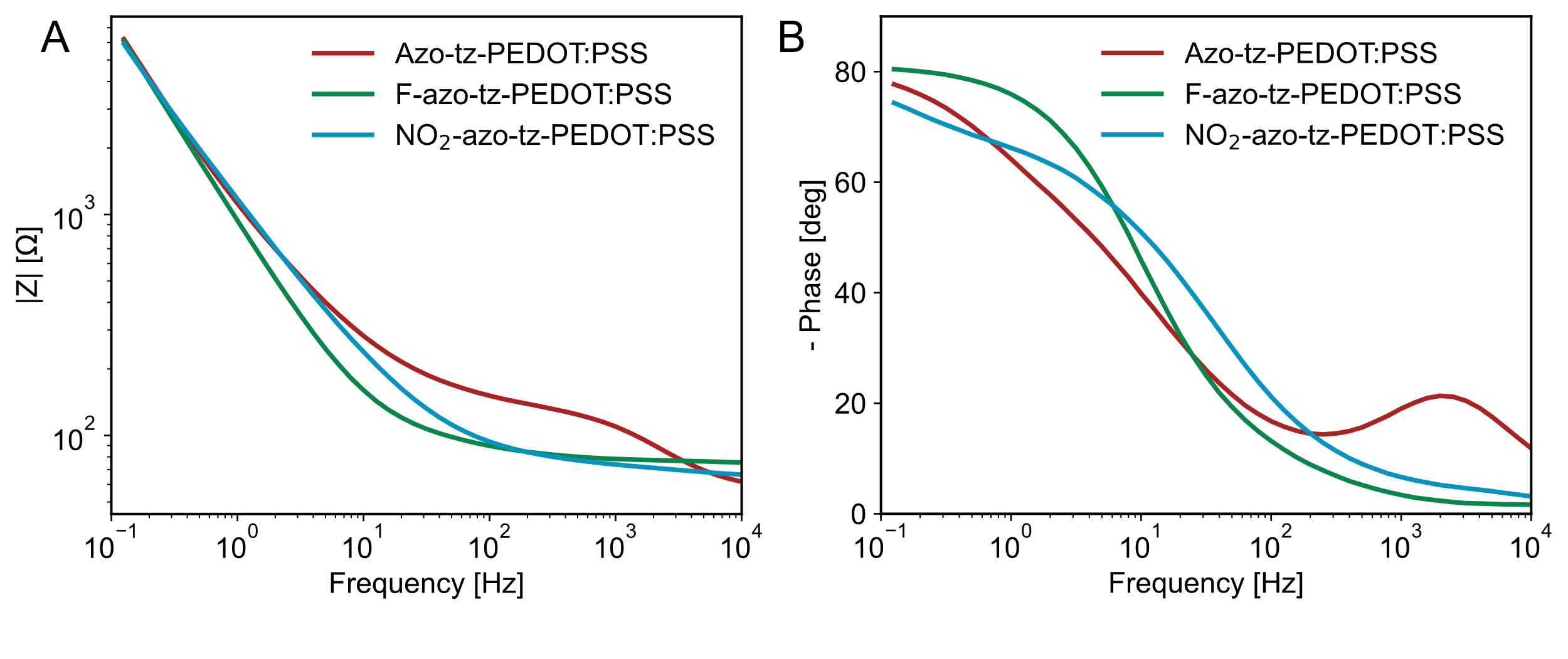


**Supplementary Figure S7. EIS analysis of the functionalized films**. Bode plots showing A) impedance amplitude and B) phase for azo-tz-PEDOT:PSS, F-azo-tz-PEDOT:PSS, and NO_2_-azo-tz-PEDOT:PSS films in their as-fabricated state. The data were fitted using the equivalent circuit model shown in **Figure 1D**.

|  | **Wavelength**  **[nm]** | **Intensity**  **[mW cm^-2^]** | **Exposure time to reach**  **azo’s optical saturation regime** **[s]** | **Exposure time to reach**  **F-azo’s optical saturation regime**  **[s]** |
| --- | --- | --- | --- | --- |
| **UV lamp** | 365 | 0.31 | 250 | 348 |
| **Arkeo 20%** | 360 - 370 | 0.92 | 88 | 120 |
| **Arkeo 60%** | 360 - 370 | 2.96 | 28 | 34 |
| **Arkeo 100%** | 360 - 370 | 4.73 | 17 | 24 |

**Table S2**. **Conditions for reaching the optical saturation regime depending on the UV source used**. Different UV light sources were used to excite the OPECTs. The time required for azo-tz-PEDOT:PSS (azo) and F-azo-tz-PEDOT:PSS (F-azo) to reach the optical saturation regime was determined by exposing the functionalized films to the reference UV lamp (as shown in **Figure 1C**). The corresponding saturation doses were calculated as 77.5 mJ cm^-2^ for azo and 107.9 mJ cm^-2^ for F-azo. Based on these dose values and the known intensities of the different illumination sources, the required exposure times for each source were subsequently calculated.


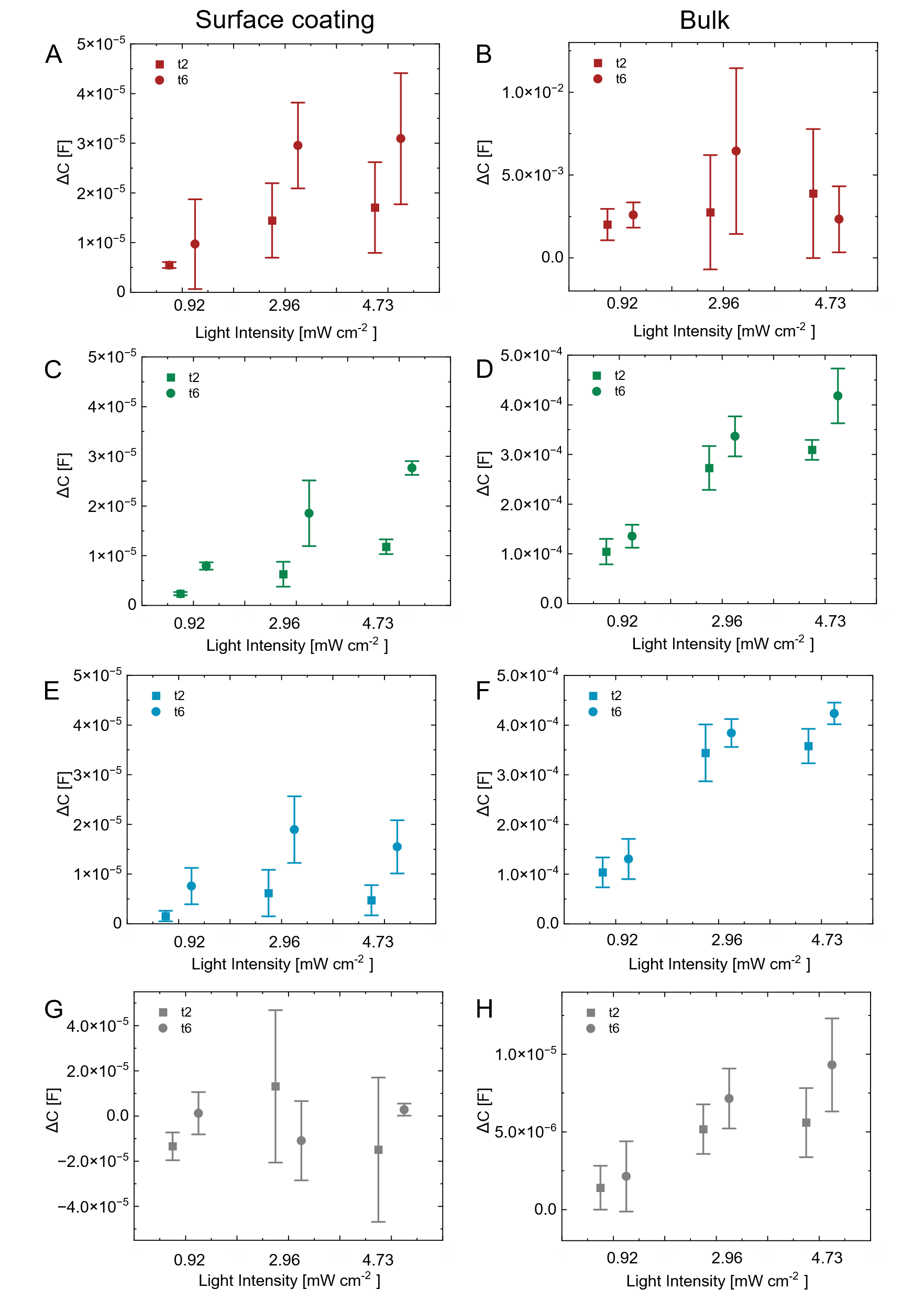


**Supplementary Figure S8. Capacitance increase in surface functionalization and bulk regions under different illumination conditions.** Capacitance changes in the surface coatings and bulk regions of films functionalized with azo (A, B), F-azo (C, D), NO_2_-azo (E, F), and non-functionalized N_3_-PEDOT:PSS (G, H). EIS spectra were acquired under dark conditions and UV illumination (wavelength: 360–370 nm; intensities: 0.92, 2.96, and 4.73 mW cm^-2^; exposure times: 2 and 6 minutes). The spectra were fitted using the equivalent circuit model to extract capacitance values. For each illumination condition, the capacitance increase was calculated as $\Delta C=C_{light}-C_{dark}$.


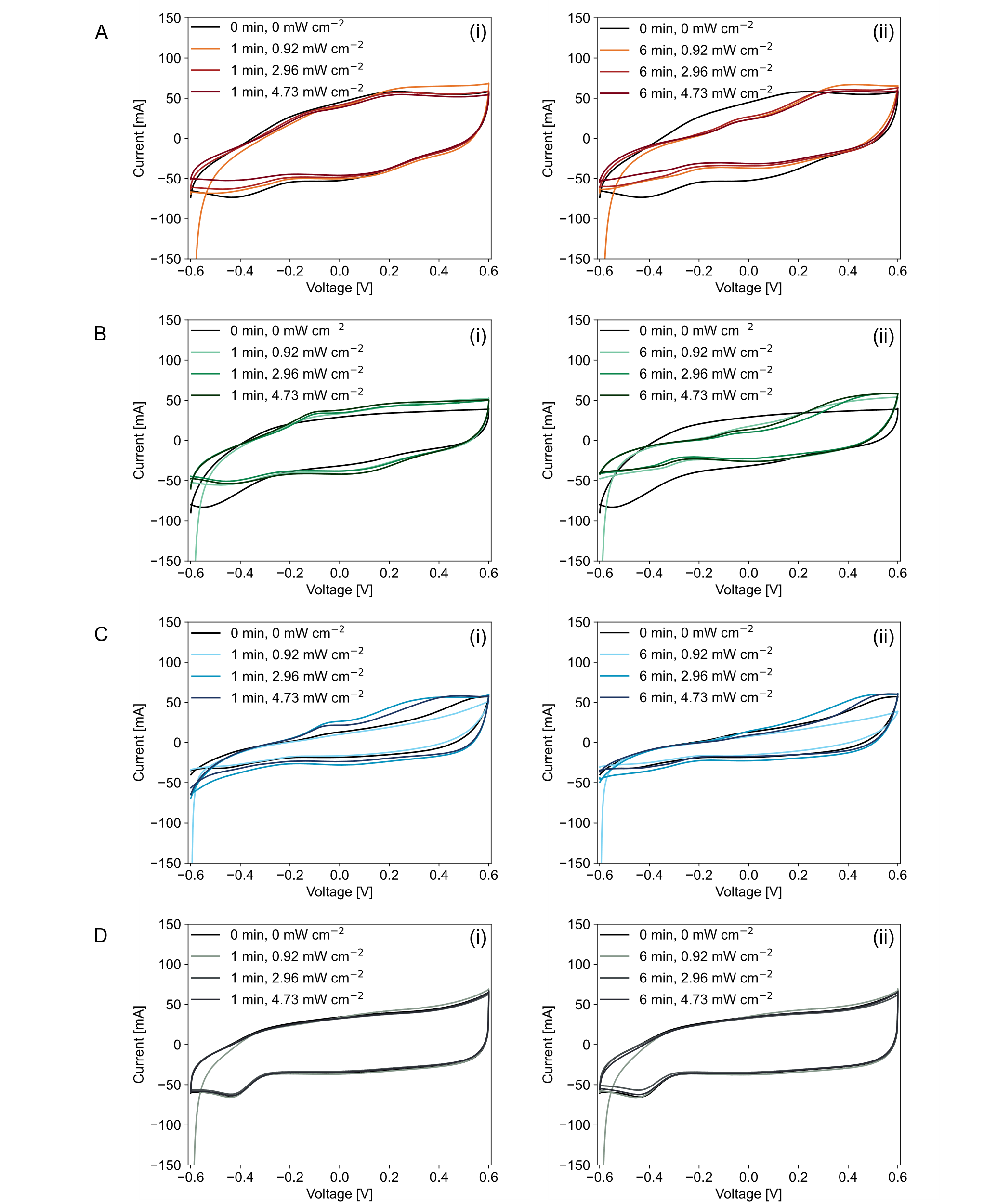


**Supplementary Figure S9. CV curves of X-azo-tz-PEDOT:PSS and N_3_-PEDOT:PSS films under dark and different illumination conditions.** Cyclic voltammetry of A) azo-, B) F-azo-, C) NO_2_-azo-tz-PEDOT:PSS, and D) N_3_-PEDOT:PSS gate electrodes recorded under dark conditions and UV illumination (wavelength: 365 nm; intensities: 0.92, 2.94, and 4.73 mW cm^-2^). Measurements were conducted after i) 60 seconds and ii) 360 seconds of prior illumination.


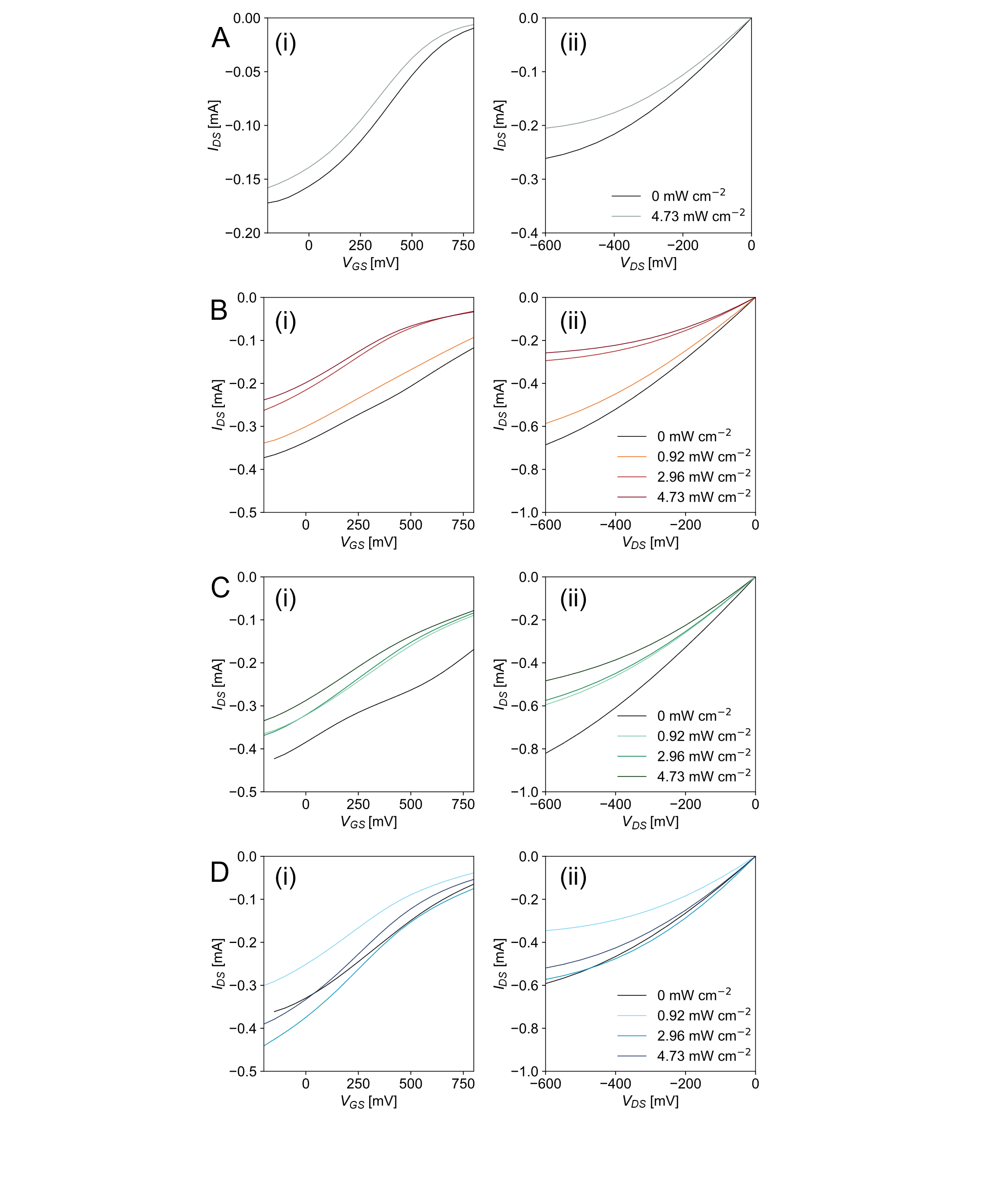


**Supplementary Figure S10. Transfer and output characteristics of the OPECTs.** Transfer (i) and output (ii) curves of A) N_3_-OPECT, B) azo-OPECT, C) F-azo-OPECT, and D) NO_2_-azo-OPECT, recorded after 2 minutes of UV illumination at different light intensities.


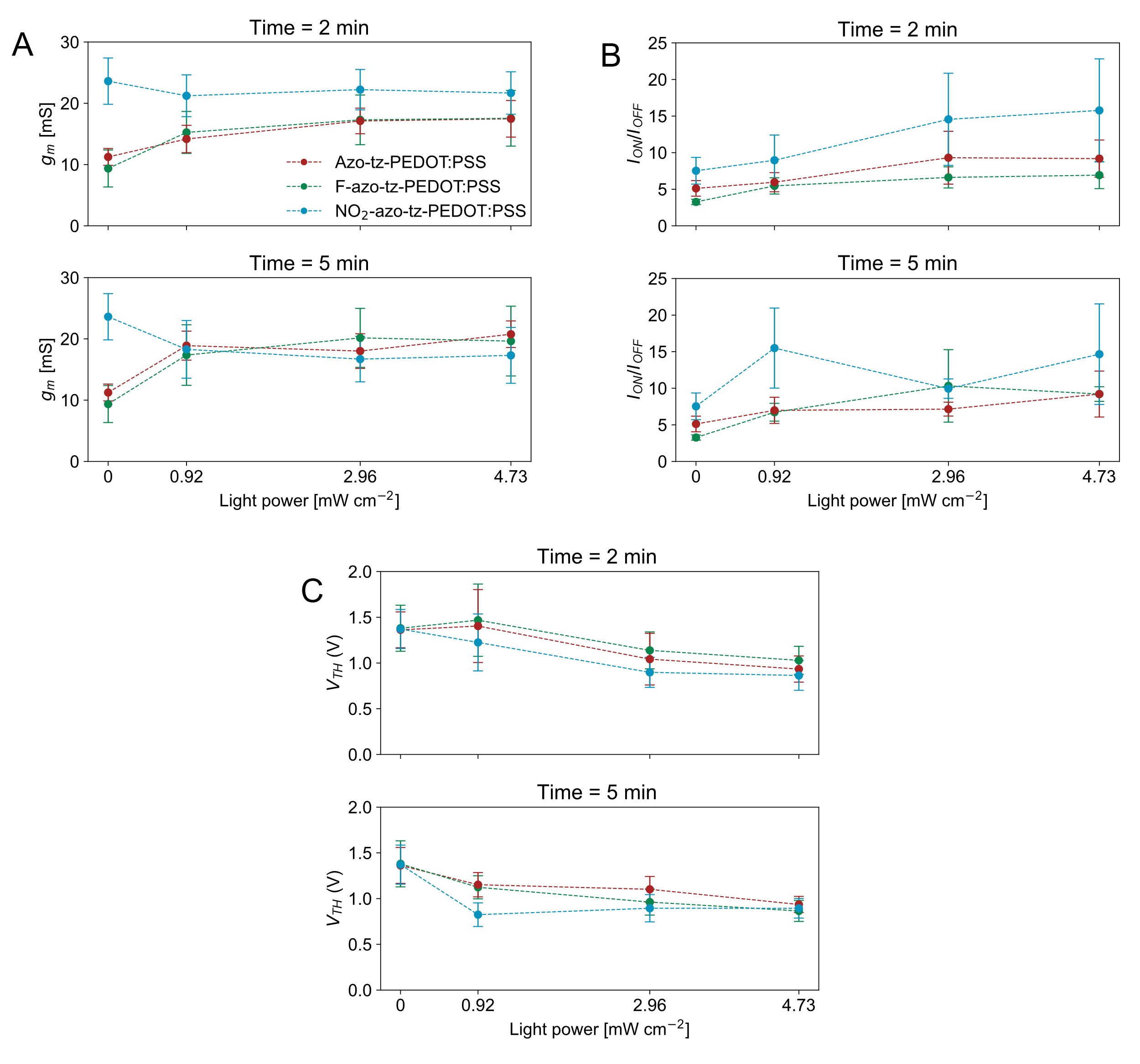


**Supplementary Figure S11. Figures of merit for the OPECTs**. A) Transconductance, B) ON/OFF current ratio and C) threshold voltage measurements after 2 and 5 minutes of illumination at different light intensities. The values obtained at 2.96 mW cm^-2^ and 4.73 mW cm^-2^ are comparable for both illumination durations.


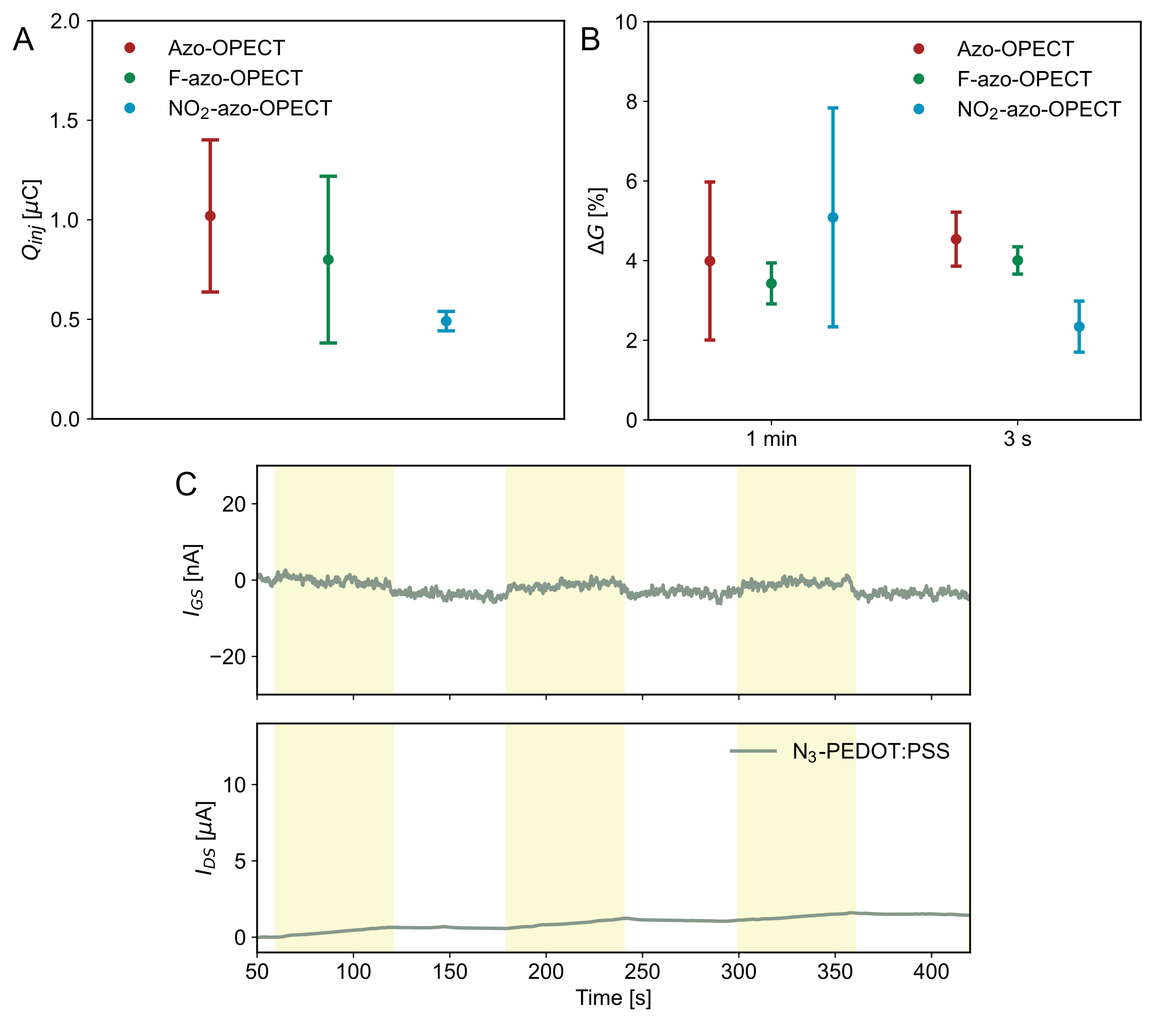


**Supplementary Figure S12. Charge injection and conductance variation under light stimulation.** A) Charge injected by the first light pulse (slow stimulation dynamics, 1-minute duration), calculated by integrating the photogenerated current over the area beneath the first pulse. The difference in injected charge between azo and F-azo is not statistically significant. B) Percentage variation in conductance ($\Delta G \left( \% \right) = \frac{G_{final}- G_{initial}}{G_{final}} \times100$) over the entire light stimulation protocol for both slow (1 minute) and fast (3 seconds) pulsing dynamics. G_final_ corresponds to the conductance at the final illumination point, and G_initial_ to the conductance immediately before illumination. C) N_3_-OPECT response to light illumination under slow pulsing conditions, as described in the Experimental Section. The lack of photocurrent confirms that no photogating occurs in the unfunctionalized N_3_-PEDOT:PSS film.


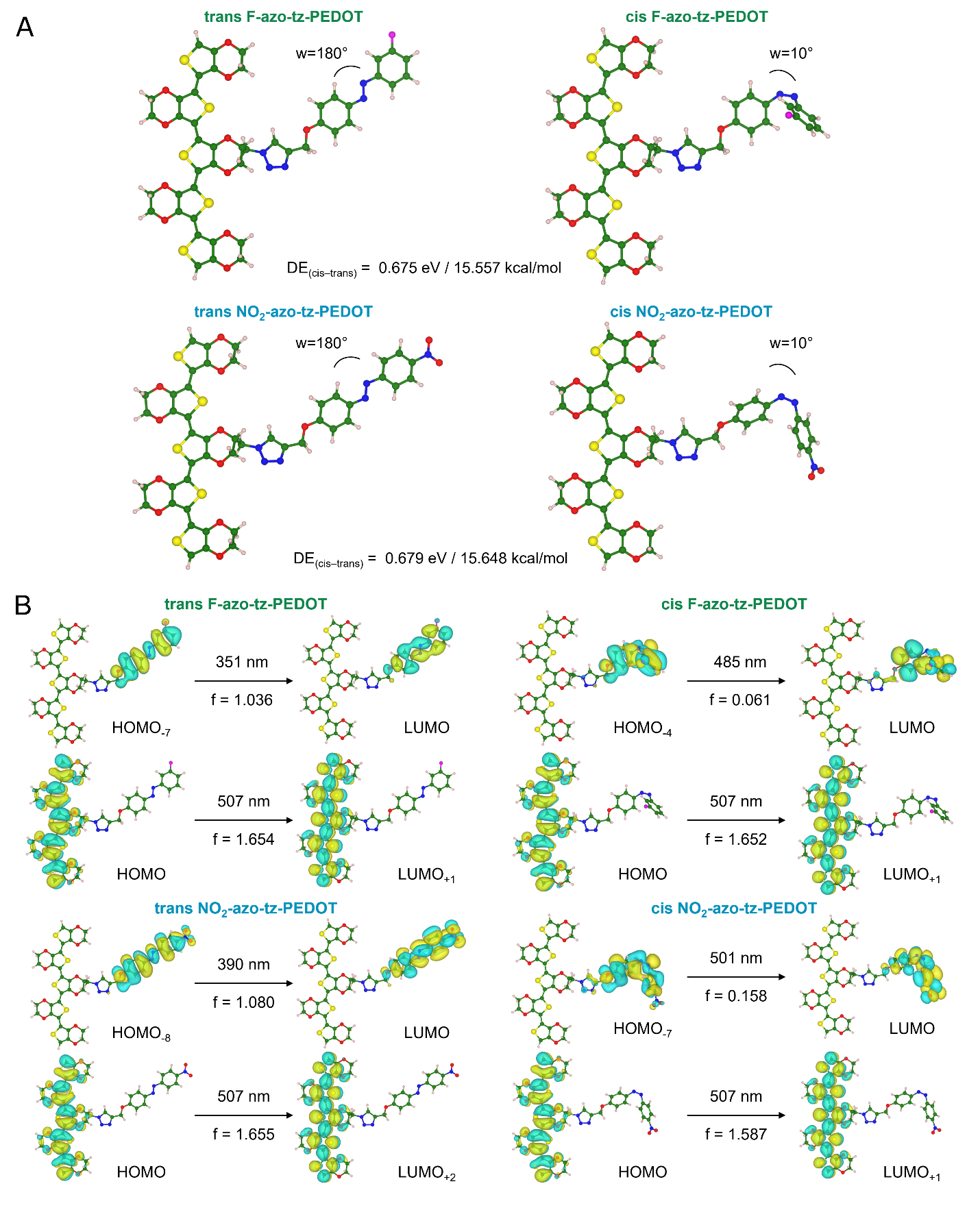


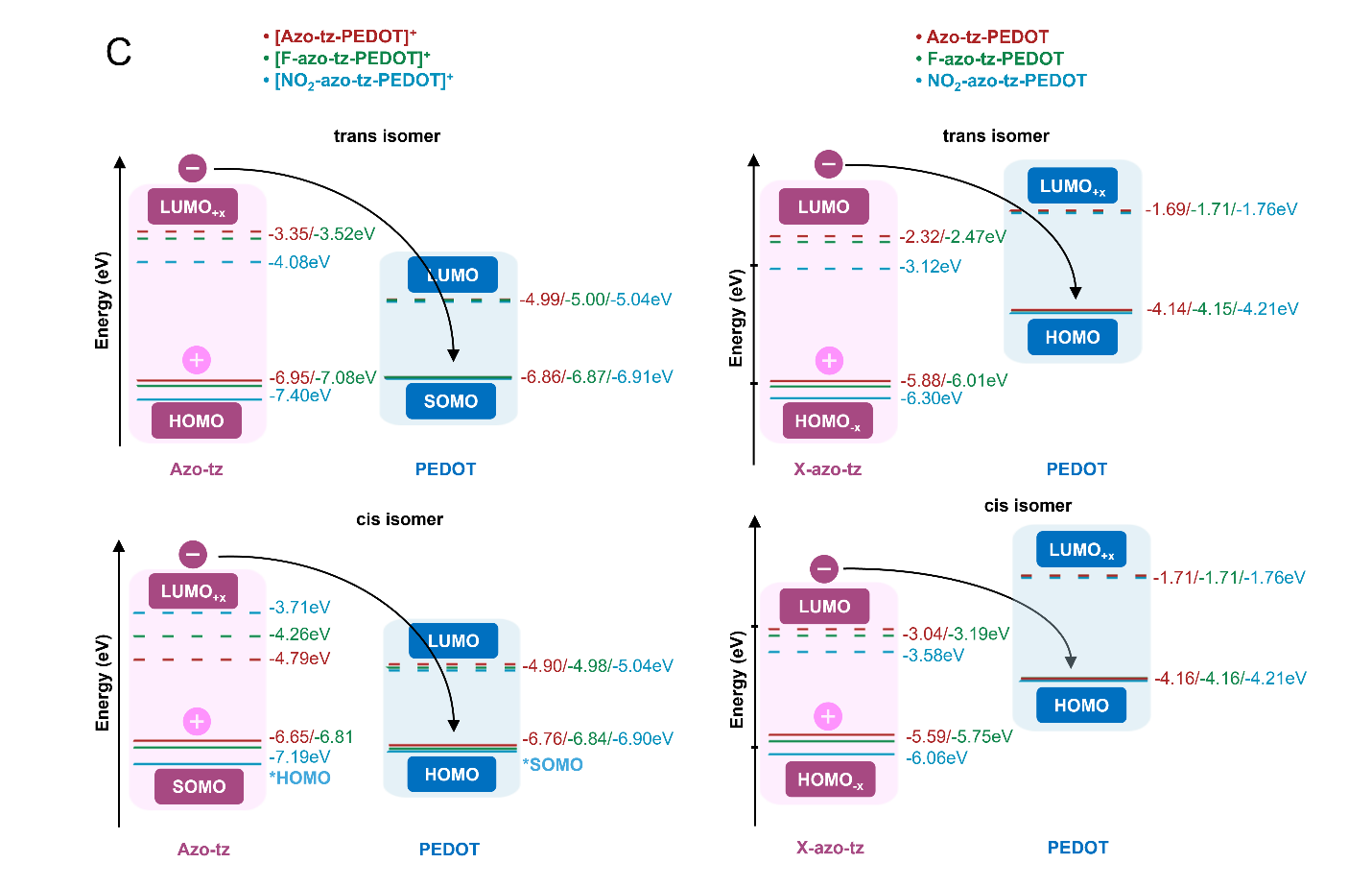


**Supplementary Figure S13. DFT and TD-DFT calculations.** A) Ground-state structures of *trans* and *cis* isomers of F-azo-tz-PEDOT and NO_2_-azo-tz-PEDOT systems, obtained at B3LYP/6-31G(d,p) level of theory. B) Electron transitions computed with TD-DFT for *trans* (left) and *cis* (right) isomers of F-azo-tz-PEDOT (top) and NO_2_-azo-tz-PEDOT (bottom) systems. The involved orbitals are shown on each structure represented in yellow and blue. Isodensity values are 0.01 eV Å^-3^. C) Energy levels of *trans* and *cis* isomers for both oxidized ([X-azo-tz-PEDOT]^+^) and neutral systems, with corresponding numerical values. HOMO/LUMO orbitals associated with the azobenzene and PEDOT moieties are highlighted in pink and blue, respectively.

**
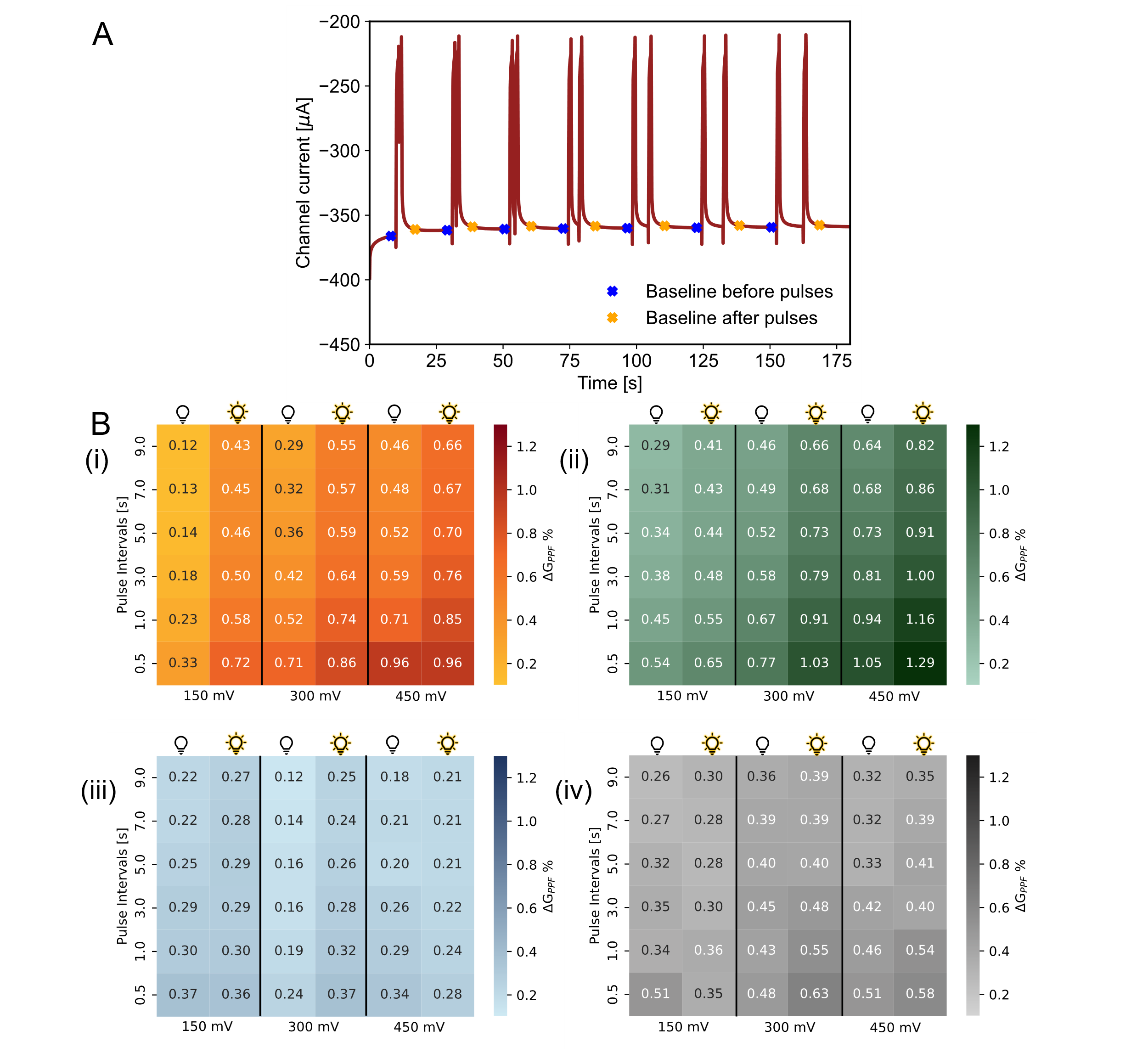
**

**Supplementary Figure S14. Pair pulsed facilitation (PPF).** A) Representative trace of the channel current recorded during a PPF experiment. A train of paired electrical voltage pulses with increasing delays was applied to the gate, with each pair separated by 20 seconds. For each pair, the PPF index was calculated by comparing conductance values 2 seconds before the first pulse (blue asterisk) and 5 seconds after the second pulse (yellow asterisk).

B) PPF heatmaps with detailed numerical values of azo (i), F-azo (ii), NO_2_-azo (iii) and N_3_-PEDOT:PSS (iv). The y-axis indicates the delay between the two pulses in each pair (0.5, 1, 3, 5, 7, and 9 seconds), while the x-axis shows the three applied V_GS_ amplitudes. For each applied V_GS_, left columns represent measurements without UV illumination, and right columns show results after UV exposure. The color map depicts the PPF index as the percentage change in conductance, with darker shades corresponding to higher facilitation.
